# High-Seas Marine Microorganism Delivers an Extract That Dampens LPS-Driven Pro-Inflammatory Signaling: *Galbibacter orientalis* Strain ROD011

**DOI:** 10.3390/md23100409

**Published:** 2025-10-18

**Authors:** Minji Kim, You-Jin Jeon, Bomi Ryu, Young-Mog Kim, Jae-Il Kim, Minkyeong Choi, Sohee Kim, Jihye Lee, Jimin Hyun

**Affiliations:** 1 Research Center for Marine Integrated Bionics Technology, Pukyong National University, Busan 48513, Republic of Korea; minjikim.pknu@gmail.com (M.K.);; 2 Department of Marine Life Science, School of Marine Biomedical Sciences, Jeju National University, Jeju 63243, Republic of Korea; 3 Department of Food Science and Nutrition, Pukyong National University, Busan 48513, Republic of Korea; 4 Department of Food Science and Technology, Pukyong National University, Busan 48513, Republic of Korea; 5 Laboratories of Marine New Drugs, Redone Technologies Co., Ltd., Jangseong-gun 57247, Republic of Korea

**Keywords:** high-seas resources, *Galbibacter orientalis* strain ROD011, deep-sea microbiome, anti-inflammatory activity, metabolomics, RAW 264.7 macrophages, zebrafish

## Abstract

An ethyl acetate extract from the deep-sea bacterium *Galbibacter orientalis* strain ROD011 (GOEE), collected from international waters, was investigated as a potential anti-inflammatory agent. In lipopolysaccharide (LPS)-stimulated murine macrophages, nitric oxide (NO) production fell by 72–87% at 5–20 µg/mL GOEE without detectable cytotoxicity. Cyclooxygenase-2 (COX-2 protein abundance decreased in a dose-dependent manner and was nearly absent at 20 µg/mL. In zebrafish embryos, survival was maintained up to 40 µg/mL, and LPS-induced signals were attenuated; the cell-death rate declined from 10 µg/mL onward, and at 20 µg/mL GOEE, reactive oxygen species (ROS) and NO decreased by 85% and 27%, respectively. To explain these effects, untargeted metabolomics with pathway enrichment and network mapping were performed in LPS-driven macrophages. Of the 58 KEGG pathways evaluated, 18 reached significance, notably purine and pyrimidine metabolism, vitamin B6 metabolism, and the one-carbon pool via folate. Coordinated shifts also involved amino-acid/tricarboxylic acid (TCA)-cycle linkages, glutathione and glyoxylate/dicarboxylate, and sphingolipid pathways. Network analysis identified hubs that were concomitantly reprogrammed. Collectively, GOEE achieved multi-level suppression of inflammatory outputs while preserving viability, and the metabolomic signature provides a mechanistic scaffold for its action. These findings nominate a deep-sea microbial extract as a promising anti-inflammatory lead and motivate fractionation and targeted validation of the highlighted metabolic nodes.

## 1. Introduction

Global competition over genetic resources has sharpened as countries operationalize access-and-benefit sharing (ABS) frameworks under the Convention on Biological Diversity [1]. The Nagoya Protocol, which entered into force on 12 October 2014, affirms national sovereignty over genetic resources and mandates fair, equitable benefit-sharing from their utilization, reshaping how bioresources are accessed and valorized [2]. Ongoing negotiations around digital sequence information (DSI) further extend the ABS conversation from physical samples to digitized genetic data, underscoring both the economic value of biodiversity-derived knowledge and the urgency of locally anchored innovation pipelines [3]. Together, these developments have intensified attention to high-seas marine resources, especially deep-sea resources.

The scope of the opportunity has been underscored by ocean metrics show that approximately 70% of Earth’s surface is ocean, and roughly 90% of that volume lies deeper than 200 m, conditions that define the deep-sea [4]. Within this context, deep-sea ecosystems represent a compelling frontier for drug discovery and functional materials. The selection pressures of high hydrostatic pressure, low temperature, and oligotrophy foster unique microbial metabolisms that yield structurally distinct molecules. Translational precedents are already emerging in various studies. For example, the HE800 exopolysaccharide (EPS) from the hydrothermal-vent bacterium *Vibrio diabolicus* has been assembled into glycosaminoglycan-mimetic biomaterials and shown efficacy in bone and skin regeneration, illustrating how extremophile-derived polymers can be engineered for clinical ends [5,6,7]. Genomic analyses have begun to resolve the biosynthetic loci underpinning these architectures, offering roadmaps for scalable production.

Anti-inflammatory activities have likewise been documented across deep-sea–linked matrices and taxa using macrophage models relevant to innate immunity. An EPS (B3-15) suppressed inducible nitric oxide synthase (iNOS) expression, nitric oxide (NO) release, and pro-inflammatory cytokines in lipopolysaccharide (LPS)-stimulated RAW 264.7 cells without cytotoxicity [8]. Refined deep-sea water-based culture medium reduced iNOS/cyclooxygenase-2 (COX-2) expression and prostaglandin output in the same model via mitogen-activated protein kinase (MAPK)/nuclear factor kappa-light-chain-enhancer of activated B cells (NF-κB) modulation [9]. Complementing these matrix-level effects, secondary metabolites from deep-sea–derived fungi have repeatedly inhibited LPS-induced NO production in RAW 264.7 macrophages, expanding the small-molecule chemical space for marine anti-inflammatories [10]. Despite these promising findings, the chemical space of therapeutic small molecules from deep-sea bacterial extracts remains largely underexplored, particularly those derived from high-seas benthic bacteria that thrive in oligotrophic, high-pressure environments. This represents a critical opportunity for discovering structurally distinct bioactive compounds.

Building on these advances, we investigated an ethyl acetate (EtOAc) extract from *Galbibacter orientalis* strain ROD011, isolated from continental-shelf sediments off 16.096898° S, 151.884181° W in the Pacific Ocean. *Galbibacter* spp. (family *Flavobacteriaceae*) was originally erected from marine sediments and now includes species recovered from deep-sea environments; recent taxonomic work also transferred *Joostella marina* to *G. orientalis* (nom. nov.), refining the genus and highlighting its marine provenance [11,12]. The sediment-associated lifestyles of these lineages suggest access to niche carbon sources and stressors that can drive uncommon metabolic chemistries [13].

In this study, the EtOAc extract of *G. orientalis* strain ROD011 (GOEE) was investigated for its protective role under LPS-triggered macrophage activation by remodeling metabolic circuits linked to inflammatory signaling. To test this, the phenotypic readouts [NO and reactive oxygen species [14]] in RAW 264.7 macrophages were profiled. Subsequently, we coupled these measurements with metabolite-set enrichment and metabolite–pathway network mapping to delineate mechanism-level effects.

In designing this work, we deliberately focused on the extract-level investigation to establish reproducible anti-inflammatory activity while capturing a global metabolic signature that could guide subsequent compound-level studies. Rather than isolating single molecules at this stage, we aimed to define GOEE’s integrated pharmacological profile across cellular and organismal systems. This approach allows us to identify tractable biochemical nodes that can later be explored through bioassay-guided fractionation and structural characterization, which are currently underway in our laboratory.

By situating a deep-sea microorganism within the evolving ABS/DSI landscape, our study aims to (i) address the scarcity of structurally diverse small-molecule anti-inflammatory leads from high-seas bacteria and ref. [15] provide a model for equitable, locally grounded valorization of national marine genetic resources.

## 2. Results

### 2.1. Deep-Sea Origin, Taxonomic Placement, and Extract Preparation of G. orientalis Strain ROD011

**Figure 1:**
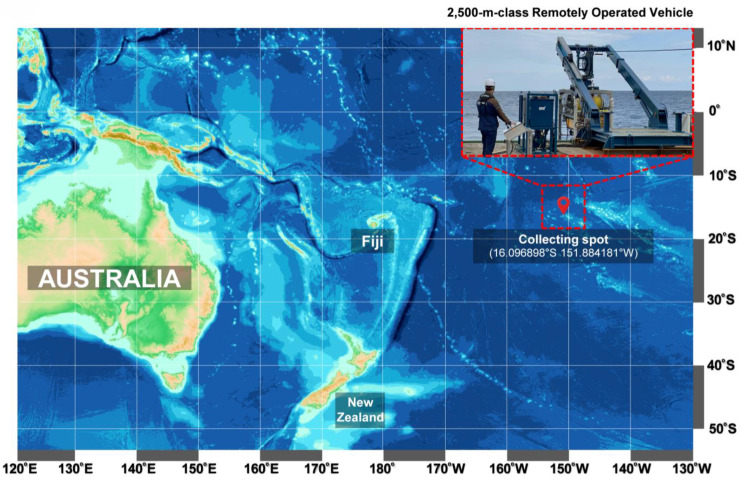
The bathymetric map of the remote submarine robot drop point for sample collection.

**Figure 2:**
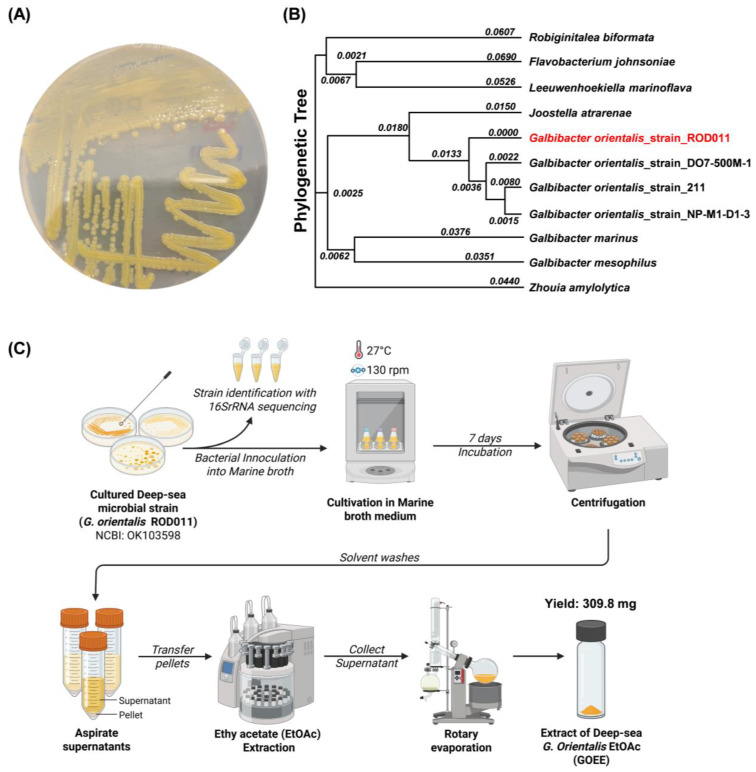
16S rRNA phylogenetic placement of *G. orientalis* strain ROD011 and Preparation of GOEE. (**A**) The colony morphology of *G. orientalis* strain ROD011 on the agar plates. (**B**) 16S rRNA–based phylogenetic tree of the deep-sea bacterium *G. orientalis* strain ROD011, which is marked in red. (**C**) Workflow for GOEE production: strain identification by 16S rRNA sequencing, inoculation into marine broth, cultivation at 27 °C and 130 rpm for 7 days, centrifugation, liquid–liquid extraction of the culture supernatant with EtOAc, and rotary evaporation to yield GOEE.

Strain ROD011 was isolated from a Western Pacific deep-sea sediment sample collected at 1480 m depth near 16.096898° S, 151.884181° W (Figure 1). Sediment was collected from the collection point used as the source for specific microbial isolation and downstream extraction. Colonies obtained after enrichment on Marine Agar 2216 displayed a uniform yellow pigmentation with circular, convex morphology and smooth surfaces, which was stable upon subculture (Figure 2A). Light microscopy revealed short rods consistent with members of the family *Flavobacteriaceae*. A representative colony was selected and designated *G. orientalis* strain ROD011. Phylogenetic placement based on nearly full-length 16S rRNA sequences confirmed this assignment (Figure 2B). The 16S rRNA gene from strain ROD011 (GenBank OK103598) aligned with reference taxa from *Flavobacteriaceae* and matched *G. orientalis* type material with 100% identity. Trees were inferred in MEGA using MUSCLE alignment, the Kimura 2-parameter distance model, and Neighbor-Joining with 1000 bootstrap replicates; the tree was midpoint-rooted, and the tip for strain ROD011 was highlighted for clarity. Together, these analyses supported the classification of the isolate as *G. orientalis* strain ROD011.

GOEE was prepared according to the workflow (Figure 2C). The cultivation of *G. orientalis* strain ROD011 in marine broth medium for seven days resulted in a light-yellow culture. Following centrifugation and isolation of the microbial pellet, extraction with EtOAc yielded the GOEE extract. The GOEE organic phase exhibited a significantly intensified, deep yellow color compared to the original light-yellow broth. This marked visual change strongly suggested that the lipophilic pigments and other medium-polarity secondary metabolites were efficiently partitioned from the microbial biomass. It is inferred that the strong organic solvent system facilitated the necessary disruption of the microbial cell walls, enabling the successful elution of these intracellular lipophilic constituents into the GOEE, thereby providing preliminary evidence for the potent activity investigated in the following experiments. GOEE was used in subsequent in vitro and in vivo assays to evaluate anti-inflammatory potential.

### 2.2. GOEE Abrogates COX-2/NO Signaling and Reprograms Inflammatory Metabolism in Macrophages

**Figure 3:**
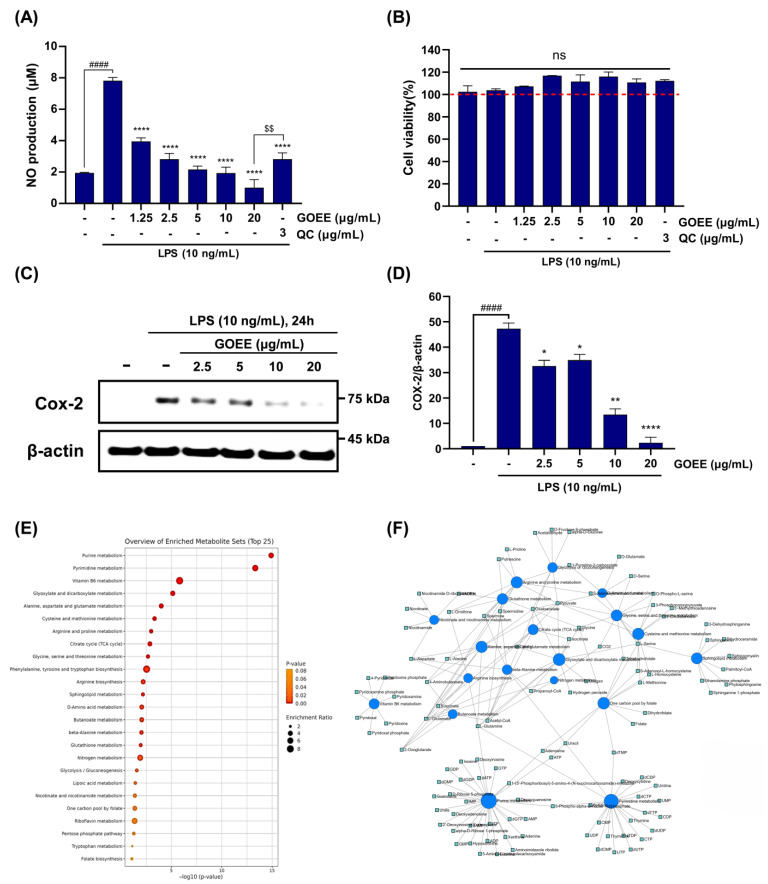
Anti-pro-inflammatory activity of GOEE in LPS-induced murine macrophages and metabolomic pathway insights from enrichment and network analyses. (**A**) GOEE suppresses LPS-induced NO production in RAW 264.7 cells in a concentration-dependent manner. Cells were pretreated with GOEE (1.25–20 µg/mL, 1 h) or quercetin (QC, 3 µg/mL; positive control) and then challenged with LPS (10 ng/mL, 24 h); nitrite levels were quantified using the Griess assay. (**B**) GOEE shows no cytotoxicity at the tested concentrations; cell viability (MTT) remained at 100% (red dashed line) with no significant differences vs. the LPS sole group. (**C**) Representative immunoblot of COX-2 in RAW 264.7 macrophages stimulated with LPS (10 ng/mL, 24 h) with or without GOEE (2.5–20 µg/mL); β-actin served as a loading control. (**D**) Densitometric quantification of COX-2 normalized to β-actin. (**E**) Metabolite-set enrichment summary (top 25) generated in MetaboAnalyst v6.0 (*Mus musculus*): *x*-axis, −log10(adjusted P); bubble size, number of matched metabolites (hits); color scale, adjusted P (FDR). (**F**) Metabolite–metabolite interaction network from the Network Explorer module; blue nodes represent identified metabolites and edges their curated interactions (HMDB/STITCH). Node size is proportional to degree centrality, highlighting putative metabolic hubs. Data are mean ± SEM. * *p* < 0.05, ** *p* < 0.01, and **** *p* < 0.0001 vs. LPS. ^####^ *p <* 0.0001 vs. the untreated control. ^$$^ *p* < 0.01 vs. QC. ns, not significant.

RAW 264.7 murine macrophages were then challenged with LPS (10 ng/mL) and were subsequently exposed to GOEE at doses of 5, 10, or 20 µg/mL (Figure 3A–D). Compared to cells treated with LPS alone, which produced robust NO, GOEE supplementation significantly reduced NO production, indicating a dose-dependent attenuation of macrophage activation (Figure 3A). NO production rates of all groups were normalized to the mean of the LPS-only control group (100%), and those were recorded as 27.67 ± 2.81% for GOEE 5 µg/mL, 24.77 ± 4.76% for 10 µg/mL, and 12.79 ± 6.71% for 20 µg/mL. The quercetin positive control (QC) showed at a 36.05 ± 5.20% in 3 µg/mL concentration, whereas LPS alone measured 100.00 ± 2.60% (Figure 3A). From these normalized means, absolute reductions relative to LPS were calculated as 72.33%, 75.23%, and 87.21% for GOEE 5, 10, and 20 µg/mL, respectively, compared with 63.95% for QC (Figure 3A). However, cytotoxicity of GOEE in murine macrophage was not detected across a 5–20 µg/mL dose range; instead, modest viability elevations were observed (Figure 3B). Taken together, GOEE exposure in macrophages suppressed LPS-induced pro-inflammatory responses without adverse cellular effects.

Expression of COX-2, a key enzyme in the NO/PGE_2_ axis during macrophage activation, was induced by LPS (10 ng/mL) and was visibly reduced when GOEE treatment was applied at 5, 10, and 20 µg/mL, while β-actin remained invariant and served as the loading control (Figure 3C). To demonstrate the inhibitory effect of GOEE distinctly, densitometric quantification was performed by dividing the COX-2 band intensity of each lane by the intensity of its matched β-actin band, yielding unitless COX-2/β-actin ratios (Figure 3D). Under LPS (10 ng/mL), the mean ± SEM ratio was 47.29 ± 1.56 a.u., establishing the reference distribution for comparison (Figure 3A). Intriguingly, this LPS-stimulated COX-2 protein expression was markedly reduced by a series of GOEE treatments (ratios of 32.64 ± 1.56 a.u. at 2.5 µg/mL, 34.92 ± 1.56 a.u. at 5 µg/mL, 13.49 ± 1.56 a.u. at 10 µg/mL, and 2.31 ± 1.56 a.u. at 20 µg/mL). These data also indicate modest attenuation at 2.5–5 µg/mL, a pronounced drop at 10 µg/mL, and near-complete suppression at 20 µg/mL (Figure 3D). These protein-level data establish that the suppression of an upstream inflammatory enzyme accompanies GOEE exposure in macrophages, providing orthogonal support for the phenotype established in the earlier assays.

### 2.3. GOEE Reprograms Inflammatory Metabolism in Macrophages

To investigate how this suppression is linked to intracellular metabolism, a metabolite-set enrichment analysis was performed (Figure 3E). Among 58 KEGG pathways analyzed, 18 were significantly enriched (FDR < 0.05). The most affected categories were purine and pyrimidine metabolism, both essential for nucleotide synthesis and inflammatory signaling. Strong enrichment was also observed in vitamin B6 metabolism and the one-carbon pool by folate, pathways that provide cofactors for amino acid conversion and methylation reactions (Figure 3E). Additional shifts were detected in amino acid metabolism (alanine/aspartate/glutamate; glycine/serine/threonine; arginine/proline), the tricarboxylic acid (TCA) cycle, glutathione metabolism, and sphingolipid metabolism, pointing to broad changes in energy, redox balance, and membrane signaling (Figure 3E). A network map was then constructed to connect these enriched pathways to specific metabolites (Figure 3F). Central nodes such as pyridoxal-5′-phosphate (vitamin B6) and folate/dihydrofolate linked amino acid transamination and one-carbon reactions, supporting the redirection of nitrogen and methyl units. Lipid mediators such as sphinganine, phytosphingosine, and dihydroceramide clustered within a sphingolipid module related to stress and apoptosis. Finally, dense subgraphs of ATP/ADP/AMP and UMP/CMP/UTP reflected remodeling of nucleotide pools under inflammatory pressure (Figure 3F).

Taken together, the results demonstrate that GOEE suppresses LPS-induced inflammation in macrophages by acting at both the protein and metabolic levels. COX-2, a pivotal pro-inflammatory enzyme, was progressively reduced and nearly eliminated at 20 µg/mL, while metabolomics revealed coordinated reprogramming of nucleotide synthesis, one-carbon cofactors, amino acid flux, and sphingolipid signaling. By aligning protein suppression with multi-pathway metabolic shifts, GOEE emerges as a deep-sea microbial extract that counteracts macrophage activation through an integrated, multi-tier mechanism.

### 2.4. GOEE Supplementation Dampened Pro-Inflammatory Signals in Zebrafish Model

**Figure 4:**
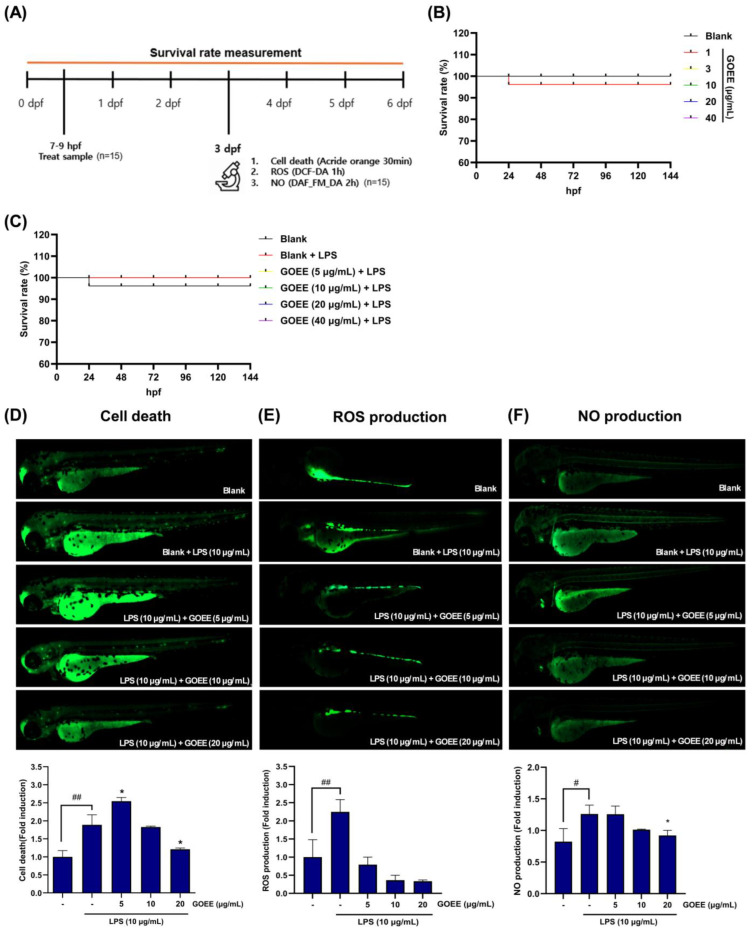
GOEE protects zebrafish larvae from LPS-evoked pro-inflammatory outputs. (**A**) Experimental timeline. Embryos were collected at 7–9 h-post fertilization (hpf), pre-exposed to GOEE, challenged with LPS, and assayed at 3 days post fertilization (dpf) for cell death (acridine orange), ROS (DCF-DA), and NO (DAF-FM DA); survival was monitored daily to 5–6 dpf (n = 15 per group). (**B**) GOEE alone does not affect larval viability. Survival of embryos/larvae exposed to GOEE (5–40 µg/mL) is indistinguishable from blank controls. (**C**) GOEE preserves viability under inflammatory stress. The survival rate remains 100% following LPS (10 µg/mL) with GOEE co-treatment (5–40 µg/mL). (**D**–**F**) Representative fluorescence images and quantification (bottom) of LPS-induced cell death ((**D**); acridine orange), ROS ((**E**); DCF-DA), and NO ((**F**); DAF-FM DA) at 3 dpf. LPS (10 µg/mL) increased each readout, and GOEE (5–20 µg/mL) reduced the signals in a concentration-dependent manner. Bars show mean ± SEM (fold induction vs. blank). * *p <* 0.05 vs. LPS group. ^#^ *p* < 0.05 and ^##^ *p* < 0.01 vs. the untreated control.

To determine the protective effect of GOEE against LPS-stimulated zebrafish, zebrafish embryos were exposed to test articles at 7–9 hpf, and survival was monitored to 6 dpf, with cell death (Acridine orange), ROS (DCF-DA), and NO (DAF-FM DA) imaged at 3 dpf (Figure 4A). Survival rate remained essentially unchanged across the dose range of GOEE exposure (1–40 µg/mL) and was not reduced when GOEE was co-administered with LPS stimulation (10 µg/mL), indicating that the in vivo dosing window used for imaging did not introduce overt developmental toxicity (Figure 4B,C).

A baseline near unity was confirmed in the blank group (1.00 ± 0.18), and a distinct induction was produced by LPS (1.89 ± 0.28) (Figure 4D). With GOEE, the mean fold-change measured 2.54 ± 0.10 at 5 µg/mL, 1.83 ± 0.02 at 10 µg/mL, and 1.21 ± 0.04 at 20 µg/mL. Relative to LPS, these values correspond to −3.2% (10 µg/mL) and −36.0% (20 µg/mL), indicating that the cell-death rate was lowered only at ≥10 µg/mL of GOEE-treated groups, with a pronounced decline at 20 µg/mL (Figure 4D). The evaluation of ROS scavenging effect by GOEE administration revealed that blank embryos averaged 1.00 ± 0.48, and LPS sole stimulation elevated ROS production to 2.25 ± 0.34; approximately a 2.25-fold rise over baseline (Figure 4E). In contrast, GOEE reduced ROS production in zebrafish to 0.79 ± 0.21 (5 µg/mL), 0.36 ± 0.14 (10 µg/mL), and 0.33 ± 0.03 (20 µg/mL), respectively. Relative to LPS, these represent −64.7% (5 µg/mL), −83.8% (10 µg/mL), and −85.2% (20 µg/mL) decreases, respectively, placing ROS well below the blank level at 10–20 µg/mL of GOEE-treated groups (Figure 4E).

In addition, LPS-induced NO production levels in zebrafish embryos significantly escalated to 1.26 ± 0.14, about a 1.53-fold increase over the blank level (0.82 ± 0.21) (Figure 4F). With GOEE treatment, NO production was 1.26 ± 0.13 (5 µg/mL), 1.01 ± 0.01 (10 µg/mL), and 0.92 ± 0.08 (20 µg/mL), which corresponded to −0.3%, −19.6%, and −26.9% respectively, establishing a dose-responsive reduction in comparison to the LPS sole group (Figure 4F). Taken together, zebrafish imaging showed maintained survival, suppressed cell death, and concerted decreases in ROS and NO within a 5–20 µg/mL window, coherently matching the cellular outcomes and the metabolic signatures associated with anti-inflammatory reprogramming under GOEE exposure.

### 2.5. The Primary Metabolite Compositions in GOEE

**Figure 5:**
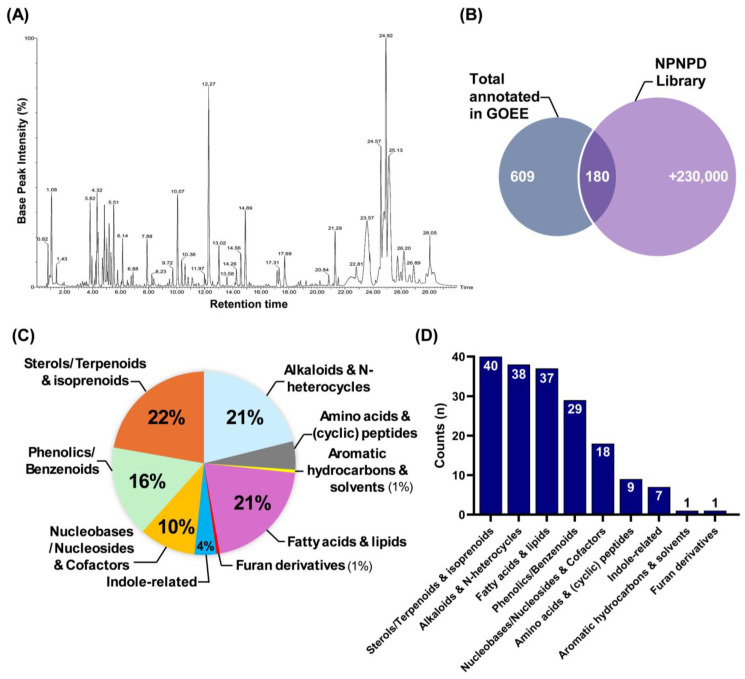
Chemical composition of the GOEE extract revealed by LC–MS and compound classification. (**A**) LC–MS base peak intensity (BPI) chromatogram of the GOEE. (**B**) Venn diagram showing the total compounds annotated in GOEE (609) and their overlap (180) with the NPNPD natural product library (>230,000 entries). (**C**) Relative proportion (%) of annotated compounds according to chemical classification. (**D**) Number of annotated compounds per chemical class.

To further characterize the chemical composition of GOEE, LC-MS/MS analysis-based metabolite profiling was conducted under reversed-chromatography conditions with a gradient mobile phase (water-acetonitrile). The BPI chromatogram revealed multiple distinct peaks across the 30 min run, highlighting the chemical complexity of the EtOAc solvent-based microorganism extract (Figure 5A). A total of 609 compound peaks were detected, of which 180 were matched to entries in the large-scale NPNPD natural product library (>230,000 compounds) (Figure 5B). The remaining majority did not align with known natural products in this reference library set. The annotated compounds encompassed a broad spectrum of chemical classes. Sterols/terpenoids and isoprenoids (40 compounds, 22%) and alkaloids and N-heterocycles (38 compounds, 21%) represented the most abundant categories, followed by fatty acids and lipids (37 compounds, 21%) and polyphenolic/benzenoids (29 compounds, 16%) (Figure 5C,D). Additional groups included nucleobases/nucleosides and Cofactors (18 compounds, 10%), amino acids and (cyclic) peptides (9 compounds, 5%), and indole-related molecules (7 compounds, 4%), whereas aromatic hydrocarbons and furan derivatives were minor components (1 compound each, 1%) (Figure 5C,D). Collectively, these data establish that the GOEE harbors a chemically diverse set of natural products spanning multiple structural categories.

## 3. Discussion

With the Nagoya Protocol foregrounding lawful access and fair benefit sharing for genetic resources under national control, the need to secure biological resources from areas beyond national jurisdiction has become more pressing [1]. The high seas are still sparsely sampled, yet they harbor organisms adapted to extreme physicochemical niches that can provide unique enzymes, small molecules, and biomaterials of scientific and industrial value. Establishing compliant pipelines for sampling, archiving, and characterization, together with clear provenance records and open metadata for associated sequence information, will expand the global knowledge base while supporting equitable benefit sharing [16]. Such stewardship ensures that discoveries from international waters translate into public value for research, conservation, and bioindustry.

Against this backdrop, a marine biological resource from the high seas was secured using a remotely operated vehicle, and its bioactivity was elucidated through pro-inflammatory response assays in this study. In LPS-stimulated RAW 264.7 cells, NO output fell by 72–87% across 5–20 µg/mL, while MTT viability stayed at 111–116% of the LPS group, showing that activity was achieved without cytotoxicity (Figure 3A,B). COX-2 protein, the enzyme that drives the NO/PGE_2_ axis in the immune system, was also lowered in a dose-dependent way and was almost absent at 20 µg/mL (Figure 3C,D). In zebrafish, survival was maintained up to 40 µg/mL, and LPS-induced signals of cell death, ROS generation, and NO production were reduced by GOEE exposure by 65–85% at 10–20 µg/mL (Figure 4A–F).

To account for these convergent outcomes, metabolomics was used to explain how these effects could arise at the systems level. Out of 58 KEGG pathways tested, 18 were significant, with leading signals including purine and pyraimidine metabolism, vitamin B6 metabolism, and the one-carbon pool by folate, plus coordinated shifts in amino-acid/TCA, glutathione and glyoxylate/dicarboxylate, and sphingolipid metabolism (Figure 3E,F). These metabolisms are tightly linked to the role of GOEE, which acts through a coordinated reshaping of nucleotide supply, cofactor availability, redox handling, and membrane-linked signaling [17,18,19,20,21]. In this framework, the fall in COX-2 protein expression and NO production was interpreted not as an isolated effect but as the visible consequence of a system whose metabolic levers had been shifted toward a less inflammatory state (Figure 3).

Compound classification analysis further clarifies how these pathway-level changes can be explained by the chemical diversity of GOEE (Figure 5). The gradient-based polar/non-polar mobile phase shift revealed the presence of a wide range of primary metabolites with varying polarity in GOEE (Figure 5A). In detail, sterols and terpenoids, alkaloids, fatty acids and lipids, phenolics, and indole derivatives together accounted for the majority of annotated compounds, whereas nucleosides, amino acid derivatives, and other minor classes contributed additional molecular diversity (Figure 5C,D). The relatively high abundance of sterols, lipids, and phenolics also reflects the selective properties of EtOAc as an extraction solvent, which preferentially enriches mid- to low-polarity metabolites (Figure 2A and Figure 5C) [22]. Such diversity is consistent with the multi-target phenotype observed in macrophages and zebrafish (Figure 3, Figure 4 and Figure 5).

Several of these annotated metabolites map directly onto the metabolic routes highlighted by enrichment analysis (Table S2). 4-methylumbelliferone and herniarin, coumarins present in GOEE, are known to suppress NF-κB and JNK-dependent inflammatory signaling, aligning with the reduced COX-2 and iNOS expression observed in our metabolomic network (Figure 3) [23,24]. Fatty acids such as γ-linolenic acid and α-linolenic acid inhibit NF-κB activation and downregulate iNOS and COX-2, consistent with the observed suppression of nitric oxide metabolism [15,25]. Pachymic acid, a triterpenoid identified in GOEE, reduces NO, PGE_2_, and cytokine production by targeting NF-κB, in line with the downshift in pro-inflammatory amino acid and TCA-linked fluxes [26]. Stigmasterol, a sterol found in the extract, modulates NF-κB/NLRP3 signaling and corresponds with the remodeling of sphingolipid metabolism [27]. δ-Tocotrienol, a vitamin E isoform also annotated in GOEE, inhibits NF-κB and explains part of the antioxidant and redox-buffering reinforcement seen in glutathione-related pathways [28]. Finally, nucleosides such as adenosine, detected in GOEE, are established activators of A2A/A2B receptors that suppress pro-inflammatory cytokines and shift macrophage polarization toward M2, consistent with the nucleotide economy and one-carbon pool reprogramming revealed by metabolomics [29].

Together, these compound-level examples provide direct evidence that the chemical constituents of GOEE map coherently onto the pathway-level shifts detected by untargeted metabolomics. Rather than presenting two separate layers of data, the metabolite annotations and metabolic networks converge to show that GOEE acts through multiple, coordinated biochemical routes that collectively redirect macrophage physiology toward a less inflammatory state.

Within this system view, nucleotide economy was read as a primary brake. Enrichment of purine and pyrimidine pathways implied that the demand–supply balance for nucleotides was reset, a change that would be expected to constrain transcriptional throughput and the synthesis of enzymes central to inflammatory signaling [19]. The large downward step in COX-2/β-actin ratios, together with the drop in cellular NO production rate, was therefore interpreted as a proximate manifestation of throttled nucleotide flow (Figure 3C,D). A cofactor axis was read in parallel. The prominence of vitamin B6 and the folate one-carbon pool, together with the central placement of PLP and folate/dihydrofolate in the network (Figure 3E,F), suggests that transamination capacity and methyl-group transfer were stabilized, supporting redox control and gene-regulatory balance under inflammatory pressure [30]. In the same vein, co-enrichment across arginine and proline, alanine/aspartate and glutamate, and glycine/serine and threonine, with links into the TCA cycle, was read as a rerouting of carbon-nitrogen flux away from iNOS-dependent NO production and toward anaplerotic fates, consistent with the stepwise decline in NO production in both cells and embryos (Figure 3A,C and Figure 4F) [14,31].

Redox buffering was interpreted as a second stabilizing pillar. Signals in glutathione metabolism and glyoxylate/dicarboxylate pathways, together with the bridging of hydrogen peroxide nodes in the network map, indicated that oxidative stress handling had been reinforced; the 65–85% decreases in zebrafish ROS at 10–20 µg/mL of GOEE treatment were therefore read as the organismal imprint of a cell-intrinsic antioxidant program rather than as collateral toxicity (Figure 3E and Figure 4E). Finally, sphingolipid clustering around sphinganine, phytosphingosine, and dihydroceramide was interpreted as a membrane-embedded control point over stress and apoptotic signaling, and the concurrent fall in acridine orange-labeled cell-death fluorescence at effective doses was taken as supportive of that membrane-signal restraint (Figure 3F and Figure 4D). Finally, the provenance of *G. orientalis* strain ROD011 from national deep-sea waters provides a tractable pathway for compliant development under ABS/DSI, aligning scientific opportunity with equitable resource governance.

Deep-sea microorganisms live under pressure, cold, and low nutrients, and their chemistry is often unlike that of land organisms [32]. In this study, such chemistry translated into a coordinated anti-inflammatory effect: a key enzyme (COX-2) was lowered, NO and ROS generations were reduced, and multiple metabolic routes, such as nucleotide, one-carbon/vitamin B6, glutathione, and sphingolipids, were shifted toward a less inflammatory state (Figure 3). Because these signals moved together while cell viability and embryo survival were maintained, deep-sea microbes can be viewed as practical sources of functional materials that act through several controllable pathways rather than a single target.

Several limits should be recognized. This study used a crude EtOAc extract, which selectively enriches mid- to low-polarity metabolites; highly polar constituents are likely underrepresented. Compound annotation relied on library matching and MS/MS inference, so many identifications remain putative and may include isomeric ambiguity; moreover, only a subset of the 609 detected features matched reference entries, leaving unknowns that could contribute to activity. The metabolomics links are associative and have not been proven causal. The biological scope was limited to one macrophage cell line and an embryonic zebrafish model, which, critically, lacked an established anti-inflammatory positive control in the in vivo setting, without disease-relevant mammalian validation. A clear path forward is apparent. Bioassay-guided fractionation should isolate the actives while the COX-2/NO readouts and the pathway signature are tracked. Future work should include the targeted tests of the highlighted nodes (nucleotide supply, one-carbon/PLP, glutathione/redox, and sphingolipids) to convert associations into mechanisms. Thereafter, extension to disease-relevant mammalian models will then allow efficacy and safety to be judged in settings where macrophage-driven inflammation is central.

## 4. Materials and Methods

### 4.1. Chemicals and Reagents

RAW 264.7 macrophages were obtained from the American Type Culture Collection (ATCC, Manassas, VA, USA). Culture media and supplements—Dulbecco’s modified Eagle’s medium (DMEM), fetal bovine serum (FBS), and penicillin–streptomycin were sourced from Welgene Inc. (Daegu, Republic of Korea). Reagents used for viability and fluorescence assays, including 3-(4,5-dimethylthiazol-2-yl)-2,5-diphenyltetrazolium bromide (MTT), dimethyl sulfoxide (DMSO), 2′,7′-dichlorofluorescein diacetate (DCF-DA), 4-amino-5-methylamino-2′,7′-dichlorofluorescein diacetate (DAF-FM DA), acridine orange, and lipopolysaccharide (LPS) were purchased from Sigma-Aldrich Inc. (St. Louis, MO, USA). Unless otherwise specified, additional cell-culture plastics and consumables were obtained from SPL Life Science Inc. (Pocheon, Republic of Korea).

### 4.2. Microbial Strain Isolation

Strain ROD011 was isolated from a sediment sample collected in May 2021 from the Western Pacific Ocean high seas (16.096898° S 151.884181° W) at a depth of 1480 m (Figure 1). An underwater robot, a 2500 m class Remotely Operated Vehicle (URI-L, Redone Technologies Co., Ltd., Jangseong-gun, Republic of Korea), was used for sampling. The sediment samples were dried in air for 24 h on a clean bench and subjected to heat shock at 55 °C for 5 min to inhibit the growth of non-spore-forming bacteria. The sediment sample was suspended in sterile seawater, and a 100 μL aliquot of the sample was plated onto marine agar 2216 (Sigma-Aldrich Inc.). The plates were incubated at 27 °C for 1–3 months, allowing morphologically distinct colonies to develop. A single yellow-pigmented colony was selected, designated as strain ROD011, and purified by repeated subculturing on Marine Agar 2216 (Figure 2A). The strain is routinely maintained on Marine Agar 2216 (Difco™, BD Diagnostics Inc., Sparks, MD, USA) and stored at −70 °C in Marine Broth 2216 (Difco™, BD Diagnostics Inc.) supplemented with 20% (*v*/*v*) glycerol for long-term preservation. The nearly complete 16S rRNA gene sequence of strain ROD011 was amplified using primers 27f and 1492r and deposited in GenBank under accession number OK103598. Sequence comparison revealed 100% identity with the type strain *G. orientalis* strain En5 (NR_044346).

### 4.3. Preparation of GOEE

GOEE was prepared according to the workflow (Figure 2C). *G. orientalis* Strain ROD011 was cultivated at 27 °C in two 2.5 L Ultra Yield Flasks, each containing 1 L of medium, shaking at 130 rpm for seven days. The medium was composed of 10 g/L soluble starch, 2 g/L yeast extract, and 4 g/L peptone, dissolved in distilled water containing 34.75 g artificial sea salt. After the seven-day incubation period, the culture broth (2 L) was harvested by centrifugation (4 °C, 10,000× *g* for 15 min) to separate the microbial biomass. The resulting microbial cell pellet was then subjected to extraction by adding pure EtOAc (100%). The mixture was vigorously agitated by shaking at 130 rpm for 24 h to facilitate cell wall disruption and elution of intracellular compounds. This extraction process was repeated three times to maximize metabolite recovery. The EtOAc-added extracts were pooled, and residual cell debris was removed by a final centrifugation (4 °C, 10,000× *g* for 15 min). EtOAc solvent was subsequently removed under reduced pressure using a rotary evaporator to yield 309.8 mg of the crude extract.

### 4.4. Phylogenetic Tree

The 16S rRNA gene sequence of *G. orientalis* strain ROD011 (NCBI accession OK103598) and reference sequences from closely related taxa in the family *Flavobacteriaceae* were retrieved from GenBank (full list in Table S1). Entire sequences (1374 bp) were aligned with MUSCLE (default parameters) as implemented in MEGA X (v11). After alignment, positions containing gaps or ambiguous bases were treated by pairwise deletion. Genetic distances were computed using the Kimura 2-parameter (K2P) model, and a Neighbor-Joining tree was inferred in MEGA. Bootstrap support was assessed with 1000 pseudoreplicates. Branch lengths are reported as substitutions per site. For display, the tree was midpoint-rooted, and the tip corresponding to strain ROD011 was highlighted (Figure 2B).

### 4.5. Conditions of UPLC-QTOF-MS/MS and Identification of Metabolites Profile

The UPLC–QTOF–MS/MS system comprised a SYNAPT XS mass spectrometer (Waters Inc., Milford, MA, USA) coupled to a Waters ACQUITY UPLC system equipped with a ZSpray™ electrospray ionization source and LockSpray™ interface. Prior to LC–MS/MS analysis, the dried GOEE was re-dissolved in 100% methanol at 1 mg/mL, vortex-mixed, sonicated for 5 min, and filtered through a 0.22 µm PTFE membrane. Chromatographic separation was performed on a Waters BEH C18 column (2.1 × 100 mm, 1.7 µm; Waters Inc.) at a flow rate of 0.3 mL/min. The injection volume was 1 µL. Solvent A consisted of water with 0.1% formic acid, and solvent B consisted of acetonitrile with 0.1% formic acid. The gradient program was as follows: 0–1 min, 95% A; 1–25 min, linear gradient to 100% B; 25–27 min, 100% B; 27.01–30 min, re-equilibration at 95% A. The mass spectrometer was operated in positive-ion mode over a scan range of 100–1200 *m*/*z*. The capillary voltage was set to 3.0 kV, the sampling cone at 40 V, the source temperature at 120 °C, and the desolvation temperature at 350 °C. The cone gas flow was maintained at 50 L/h, and the desolvation gas flow at 600 L/h. Data were acquired in MSe continuum mode with two functions: low-energy trap collision energy (6 V) and high-energy ramp trap collision energy (20–45 V).

Accurate precursor and fragment spectra were processed using MassLynx software v4.2 (Waters Inc.). Profiles were preprocessed by subtracting solvent blanks and aligning retention times to a pooled QC run. Peak detection used an absolute intensity threshold of 2000 counts, a retention time window of 0–30 min, and an adduct filter including [M+H]^+^. A combined feature matrix was assembled to ensure peak correspondence across samples, with absent features recorded as zero (no imputation applied).

To minimize interference from medium-derived compounds, LC–MS/MS data were pre-filtered using a custom reference library constructed from the known composition of Marine Broth 2216 (Difco™, BD Diagnostics Inc.). The library contained 28 representative amino acids, peptides, organic acids, carbohydrates, vitamins, and minor lipids typically detected in uninoculated media. Features matching library entries (*m*/*z* ± 5 ppm, RT ± 0.05 min, MS/MS similarity ≥ 0.8) were automatically flagged and excluded through the UNIFI™ software v1.9 SR4 (Waters Inc.) library-filtering module, following the background-subtraction precedent described by the previous literature [33]. This procedure enabled retention of microbially derived features while computationally removing medium-borne background peaks.

Metabolic constituents of GOEE were tentatively annotated using UNIFI™ software (Waters Inc.) by matching exact *m*/*z* values, retention times, and fragmentation patterns to curated reference files for compounds reported from the extract (Table S2). The filtered feature list was then processed in UNIFI™ (Waters Inc.) for tentative metabolite identification using a tiered-matching workflow combining exact mass (±5 ppm), retention-time tolerance (±0.05 min), and fragment pattern similarity (cosine ≥ 0.8) criteria. Each feature was searched against an in-house library of known natural products and subsequently cross-referenced to the NCI Program for Natural Products Discovery (NPNPD) prefractionated library [34]. The NPNPD dataset was accessed through the U.S. National Cancer Institute Developmental Therapeutics Program (DTP) collaborative repository (https://dctd.cancer.gov/programs/dtp/organization/npb/npnpd) (accessed on 1 September 2025) via a formal data-sharing request (dtpmail: dtpinfo@nih.gov). Metadata, including fraction identifiers, molecular formulas, and LC–MS/MS reference spectra, were used for spectral cross-validation following the hierarchical matching protocol [34]. Features with no corresponding matches in either the medium-compound or NPNPD libraries were considered putatively novel metabolites derived from *G. orientalis* strain ROD011. All raw and processed LC–MS/MS data, including the Marine Broth 2216 compound library, are archived in the public repository cited in the Data Availability Statement to enable independent verification and re-analysis.

### 4.6. Metabolite-Based Pathway Enrichment and Interaction Network Analysis

A list of confidently identified metabolites from the untargeted LC-MS experiment, including normalized relative intensities and group labels, was uploaded to MetaboAnalyst (version 6.0; https://www.metaboanalyst.ca/MetaboAnalyst/) (accessed on 16 August 2025) [35]. Compound identifiers were mapped using the built-in Compound ID conversion tool, prioritizing KEGG Compound IDs; unmatched synonyms were manually resolved, and duplicates were collapsed. The organism was set to *Mus musculus*. Pathway over-representation was assessed against KEGG pathways using the hypergeometric test, and pathway topology was evaluated by degree centrality to estimate node impact. Multiple testing was controlled by the Benjamini–Hochberg procedure, and statistical significance was defined as FDR < 0.05. Bubble plots were generated within MetaboAnalyst, with pathway impact on the *x*-axis, −log10(FDR) on the *y*-axis, bubble size proportional to impact, and color indicating significance.

The same metabolite list was analyzed using the Network Explorer module in MetaboAnalyst v6.0 to construct a Metabolite–Metabolite Interaction Network based on the integrated HMDB/STITCH knowledge base. After node mapping, edges were filtered using a confidence cutoff of ≥0.5. The network was visualized with the iGraph force-directed layout. Node centralities (degree and betweenness) were computed to highlight hubs, and communities were inspected to annotate functionally coherent modules. Final network graphics were exported directly from MetaboAnalyst at publication resolution.

### 4.7. In Vitro Evaluation of the Anti-Inflammatory Potential of GOEE Using LPS-Induced RAW 264.7

#### 4.7.1. Cell Culture and Evaluation of Cytotoxicity of GOEE

The murine macrophage—RAW 264.7 cells were cultured in DMEM containing 10% FBS and 1% P/S at 37 °C in a 5% CO_2_ humidified incubator (Sanyo Electric Co., Ltd., Tokyo, Japan). The cytotoxicity of GOEE was assessed by the MTT assay [36]. RAW 264.7 cells were seeded into a 96-well plate at a density of 1 × 10^5^ cells per well and incubated at 37 °C with 5% CO_2_ for 24 h. Subsequently, the cells were exposed to various concentrations of GOEE (1.25, 2.5, 5, 10, and 20 μg/mL) or QC as a positive control (3 μg/mL), and the cells were stimulated with LPS (10 ng/mL) and incubated for another 24 h. For statistical robustness, all cytotoxicity experiments were performed in triplicate biological replicates. After the 24 h exposure to the samples, 20 μL of 5 mg/mL MTT reagent dissolved in PBS was added to each well. The plate was then incubated at 37 °C for 1 h at 5% CO_2_. Following incubation, the solution was carefully aspirated from all the wells, and 100 μL of DMSO was added to dissolve the formazan crystals that had formed. The absorbance of each well was subsequently measured at 540 nm using a Synergy™ HTX microplate reader (Agilent Technologies Inc., Santa Clara, CA, USA).

#### 4.7.2. Evaluation of LPS-Induced NO Production

The inhibitory effect of GOEE on NO production was measured using the Griess assay [37]. RAW 264.7 cells were seeded into a 96-well plate at a density of 1 × 10^5^ cells/mL and incubated at 37 °C with 5% CO_2_ for 24 h. Subsequently, the cells were divided into the following experimental groups: (1) Control (Vehicle), (2) LPS alone (10 ng/mL), (3) GOEE + LPS, and (4) Positive control (3 μg/mL) + LPS. Cells were exposed to various concentrations of GOEE (1.25−20 μg/mL), and the cells were stimulated with LPS (10 ng/mL) and incubated for another 24 h. After the 24 h exposure to samples, equal volumes of the cell culture medium and Griess reagent were combined in a 96-well plate and incubated for 10 min. Then, the absorbance was measured at 540 nm using a Synergy™ HTX microplate reader (Agilent Technologies Inc.).

#### 4.7.3. Western Blotting

Western blotting was performed to quantify key inflammatory mediators. RAW 264.7 cells were seeded in 6-well plates (1 × 10^5^ cells/well) and cultured for 24 h, pretreated with GOEE (2.5, 5, 10, or 20 µg/mL) for 1 h, then stimulated with LPS (10 ng/mL) and incubated for an additional 24 h. Whole-cell lysates were prepared in RIPA buffer (89901, Thermo Fisher Scientific Inc., Waltham, MA, USA) and protein concentrations determined using the Pierce BCA assay. Equal protein amounts were mixed with sample buffer, heated at 100 °C for 5 min, resolved by 12% SDS-PAGE, and transferred to 0.45 µm nitrocellulose membranes. Membranes were blocked with 5% skim milk in TBST (room temperature, 2 h), probed with primary antibodies to the indicated targets, followed by HRP-conjugated secondary antibodies, and developed using an enhanced chemiluminescent substrate (RPN2105). Fluorescent signals were captured on a FUSION SOLO imaging system Vilber Lourment system (Vilber Lourmat Deutschland GmbH, Wielandstrasse 2, Eberhardzell, Germany) and band intensities quantified in ImageJ v1.54p (NIH, Bethesda, MD, USA) after normalization to β-actin.

### 4.8. In Vivo Evaluation of the Anti-Inflammatory Potential of GOEE Using Zebrafish

#### 4.8.1. Maintenance of Zebrafish

Adult zebrafish (*Danio rerio*) were obtained from a licensed supplier (Jeju Aquarium Inc., Jeju, Republic of Korea) and maintained as described previously [38]. Fish were housed in 3 L acrylic tanks at 28.5 °C on a 14:10 h light–dark cycle with conditioned, aerated water, and fed TetraMin^®^ flake and live brine shrimp three times daily, six days a week. Spawning was triggered by lights-on in the morning; fertilized embryos were collected within 30 min, staged, and allocated to experiments.

#### 4.8.2. Survival Rate Analysis in Zebrafish

Zebrafish embryos at 7–9 h post-fertilization were arrayed in 12-well plates (15 per well) and pre-exposed to GOEE (5, 10, or 20 μg/mL) for 1 h; LPS (10 μg/mL) was then added and cultures maintained to 24 hpf, after which survival was recorded daily from 1 to 5 day post-fertilization (dpf). The survival rate analysis was performed using independent biological replicates (n = 3).

#### 4.8.3. Evaluation of LPS-Induced Cell Death, NO Generation, and ROS Generation in Zebrafish

Fluorescence images analysis for cell death, nitric oxide (NO), and reactive oxygen species using LPS-evoked zebrafish embryos was adapted from those described by a previous study [39]. At 3 dpf, morphologically normal larvae were transferred into embryo medium in 12- or 24-well plates at five larvae per well and allowed to equilibrate for 20 min at 28 °C. To test protection against inflammation, larvae were pre-exposed to GOEE at 5, 10, or 20 µg/mL for 1 h, after which LPS (10 µg/mL) was added to the same wells. The experiment included the following four core groups for comparison: (1) Vehicle, (2) LPS alone, (3) GOEE + LPS, and (4) Positive control (3 μg/mL) + LPS. Vehicle (0.01% DMSO contained DMEM) and LPS-only controls were included on each plate. All fluorescence image analyses were conducted with independent biological replicates (n = 3). Unless otherwise specified, all incubations were performed in the dark at 28 °C, larvae were rinsed three times in fresh embryo medium between steps, and anesthesia with 0.016% (*v*/*v*) tricaine preceded imaging. For cell-death measurements, larvae exposed to LPS for 24 h were stained with acridine orange at 7 µg/mL for 30 min. For NO measurements, larvae were incubated with DAF-FM DA at 5 µM for 1 h, allowing de-esterification during the final rinse to minimize background. For ROS measurements, larvae were first challenged with LPS for 24 h and then stained with DCF-DA at 20 µM for 1 h. Fluorescence images were acquired on a Lionheart FX microscope controlled by Gen5 v3.03 (Agilent Technologies Inc.) using appropriate filter sets and identical exposure, gain, and binning across groups to avoid saturation. And the mean fluorescence intensity was quantified in ImageJ (NIH).

### 4.9. Statistical Analysis

For antioxidant and anti-inflammatory activity, dates are presented as the mean ± standard error of the mean, and the means of each treatment were compared using one-way ANOVA from GraphPad PRISM Version 10 (GraphPad Software Inc., San Diego, CA, USA). Significance levels were denoted as *^#^ p* < 0.05, *^##^ p* < 0.01, and *^###^ p* < 0.001 compared to the control, and those compared with the LPS group are denoted as * *p* < 0.05, ** *p* < 0.01, and *** *p* < 0.001.

## 5. Conclusions

This work identifies the deep-sea bacterium *G. orientalis* strain ROD011 as a promising anti-inflammatory lead and proposes a coherent systems-level mechanism of action. Across two complementary models—LPS-stimulated RAW 264.7 macrophages and zebrafish embryos—GOEE suppressed hallmark inflammatory outputs (NO, ROS, acridine-orange cell death) while preserving cell and organismal viability. Concordantly, COX-2 abundance was markedly reduced. Untargeted metabolomics linked these phenotypes to concerted remodeling of metabolic circuits, with enrichment in nucleotide (purine/pyrimidine) metabolism, one-carbon/folate and vitamin B6 pathways, amino-acid/TCA coupling, glutathione-centered redox handling, and sphingolipid signaling. Together, these findings support a model in which GOEE retunes nucleotide demand, cofactor balance, redox buffering, and membrane-linked cues to restrain inflammatory activation without overt toxicity. The implications are twofold. Scientifically, the data argue for multi-node metabolic control—rather than single-target inhibition—as a tractable route to modulate innate immune signaling. Practically, the provenance of strain ROD011 highlights deep-sea microbes within national waters as viable, responsibly developable sources of bioactive materials.

## Data Availability

Data is contained within the present paper. *G. orientalis* strain ROD011 genomic sequence has been deposited in the NCBI genome database under ID OK103598. Also, the raw mass spectrometry data and processed results have been uploaded to [https://www.dropbox.com/scl/fo/7qxqc2o71o25vq4sqnb1e/AIW7yGthL83aPc1mrEnFzw0?rlkey=txvzzl8ot5l808nhl5asmq8d1&dl=0] (accessed on 15 October 2025).

## Supplementary Materials

The following supporting information can be downloaded at: https://www.mdpi.com/article/10.3390/md23100409/s1. Table S1: The reference sequences from closely related taxa in the family Flavobacteriaceae from GenBank; Table S2: The metabolite profile in GOEE.
