# Supplementary material for: High-Seas Marine Microorganism Delivers an Extract That Dampens LPS-Driven Pro-Inflammatory Signaling: Galbibacter orientalis Strain ROD011
Source: Mar Drugs. 2025 Oct 18;23(10):409. doi: 10.3390/md23100409 (PMC12565332; doi:10.3390/md23100409)
Supplement: Supplementary file 1 [file marinedrugs-23-00409-s001.zip › (240520) polyphenol msms data.pptx]

## Slide 1
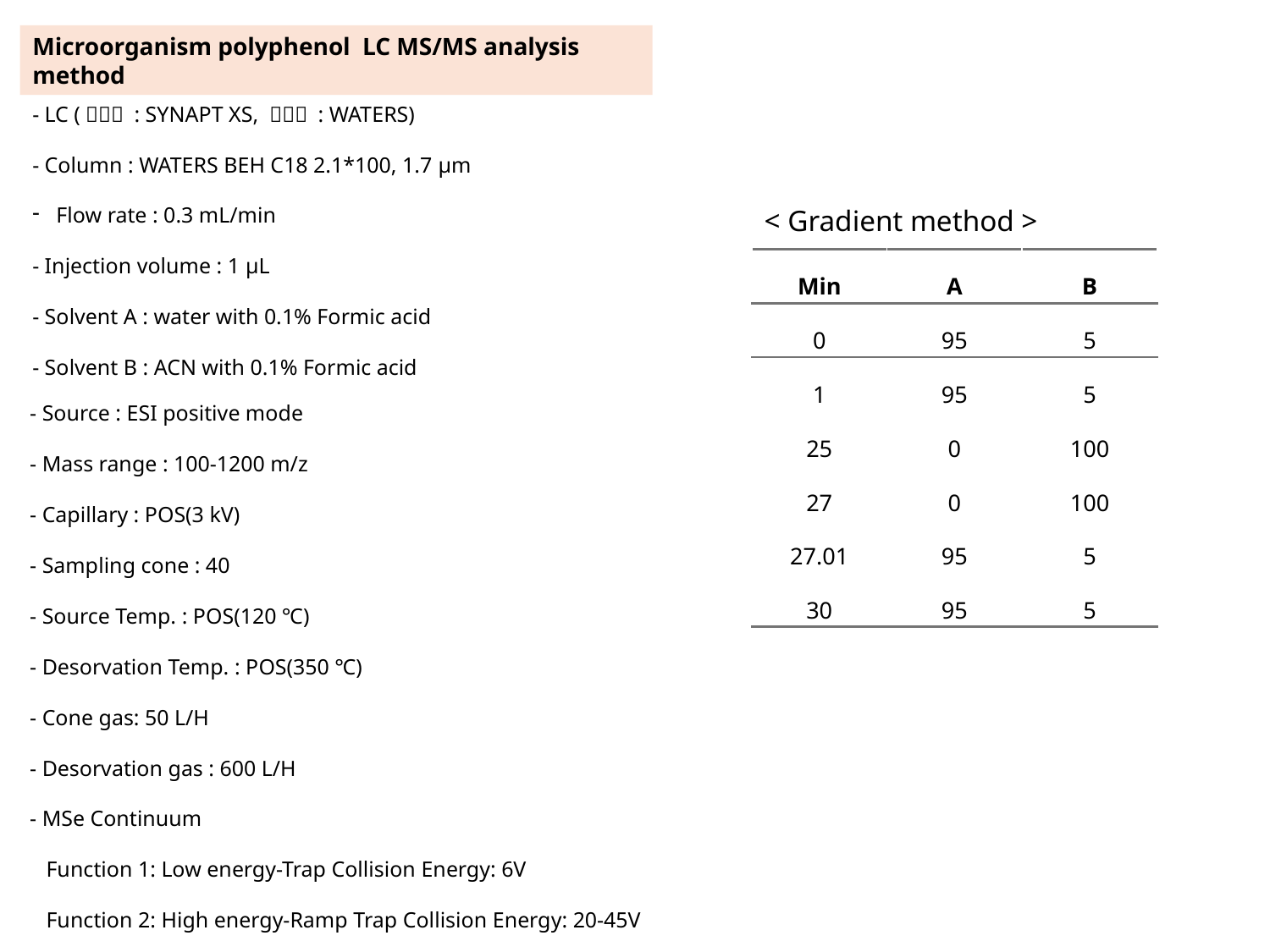

Microorganism polyphenol LC MS/MS analysis method
- LC (모델명 : SYNAPT XS, 제작사 : WATERS)
- Column : WATERS BEH C18 2.1*100, 1.7 μm
Flow rate : 0.3 mL/min
- Injection volume : 1 μL
- Solvent A : water with 0.1% Formic acid
- Solvent B : ACN with 0.1% Formic acid
< Gradient method >
| Min | A | B |
| --- | --- | --- |
| 0 | 95 | 5 |
| 1 | 95 | 5 |
| 25 | 0 | 100 |
| 27 | 0 | 100 |
| 27.01 | 95 | 5 |
| 30 | 95 | 5 |
 - Source : ESI positive mode
 - Mass range : 100-1200 m/z
 - Capillary : POS(3 kV)
 - Sampling cone : 40
 - Source Temp. : POS(120 ℃)
 - Desorvation Temp. : POS(350 ℃)
 - Cone gas: 50 L/H
 - Desorvation gas : 600 L/H
 - MSe Continuum
 Function 1: Low energy-Trap Collision Energy: 6V
 Function 2: High energy-Ramp Trap Collision Energy: 20-45V

## Slide 2
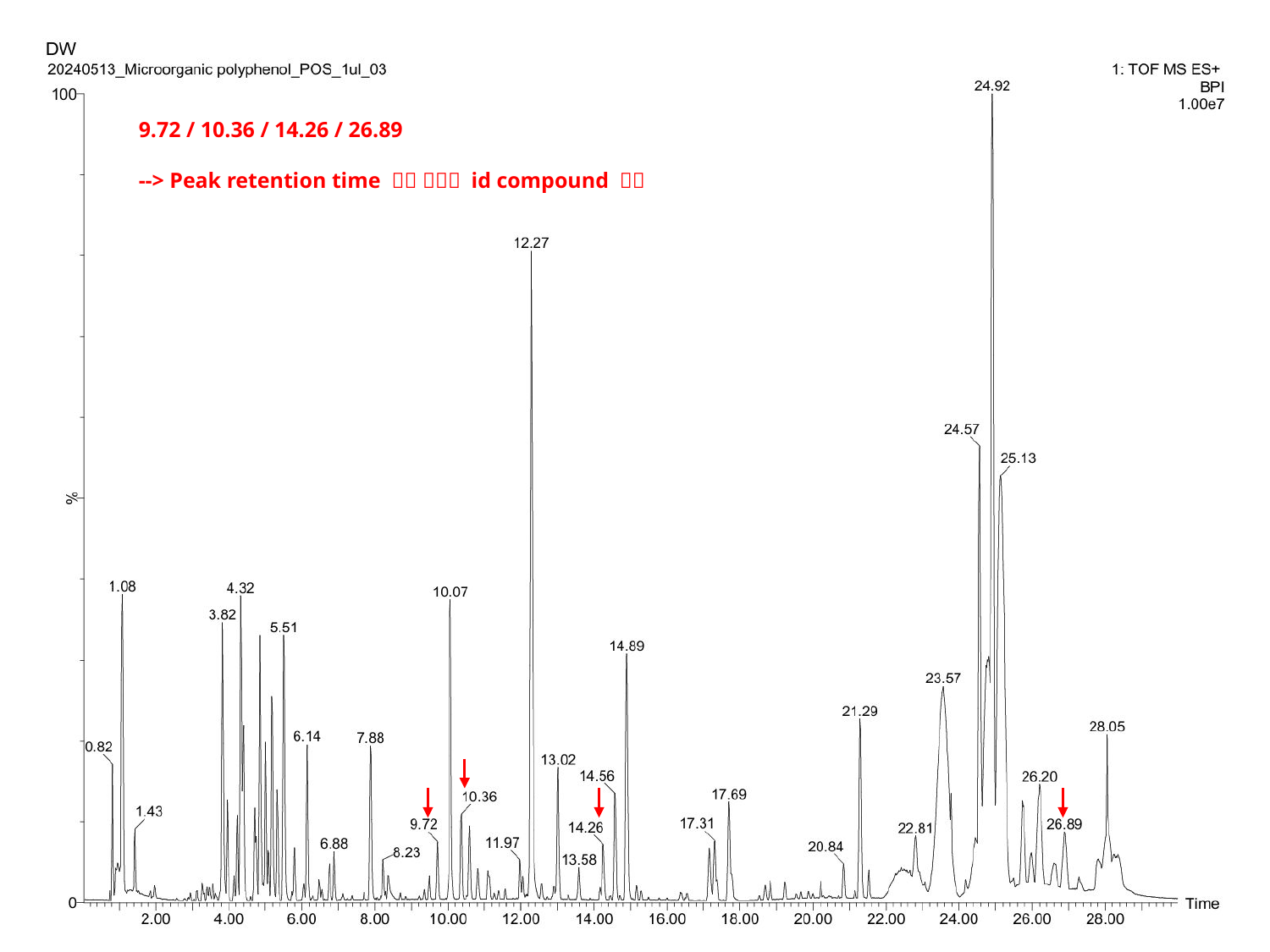

9.72 / 10.36 / 14.26 / 26.89
--> Peak retention time 이랑 겹치는 id compound 없음
