# Supplementary material for: High-Seas Marine Microorganism Delivers an Extract That Dampens LPS-Driven Pro-Inflammatory Signaling: Galbibacter orientalis Strain ROD011
Source: Mar Drugs. 2025 Oct 18;23(10):409. doi: 10.3390/md23100409 (PMC12565332; doi:10.3390/md23100409)
Supplement: Supplementary file 1 [file marinedrugs-23-00409-s001.zip › Supplementary Tables.pdf]

Supplementary data

Supplementary Table S1. The reference sequences from closely related taxa in the family *Flavobacteriaceae* from GenBank.

#	Query label	GenBank accession	Note / status
1	*Galbibacter orientalis* strain ROD011	OK103598	Type strain for 16S rRNA
2	*Galbibacter orientalis* strain NP-M1-D1-3	PV804097.1	Type strain for 16S rRNA
3	*Galbibacter orientalis* strain 211	OM056909.1	Type strain for 16S rRNA
4	*Galbibacter orientalis* strain DO7-500M-1	OK560253.1	Type strain for 16S rRNA
5	*Galbibacter mesophilus*	AB681493.1	Type strain for 16S rRNA
6	*Galbibacter marinus* strain ck-I2-15	NR_108285.1	Type strain for 16S rRNA
7	*Joostella atrarenae* strain M1-2	GQ872420.1	Type strain for 16S rRNA
8	*Zhouia amylolytica* strain HN-171	DQ423479.1	Type strain for 16S rRNA
9	*Leeuwenhoekiella marinoflava* strain LMG 1345	AB680577.1	Type strain for 16S rRNA
10	*Robiginitalea biformata* strain HTCC2514	NR_074601	Type strain for 16S rRNA
11	*Flavobacterium johnsoniae* strain LB-D	AB495173	Type strain for 16S rRNA

Supplementary Table S2. The metabolite profile in GOEE.

m/z	compound	class_expanded	Natural product
**101.0598**	δ-Valerolactone	Fatty acids & lipids	
**101.0713**	1-Nitrosopyrrolidine	Alkaloids & N-heterocycles	O
**102.0916**	Isovaleramide	Amino acids & (cyclic) peptides	
**103.0752**	3-Methylbutanoic acid	Fatty acids & lipids	O
**106.05**	L-(-)-Serine	Amino acids & (cyclic) peptides	O
**107.0491**	Benzaldehyde	Phenolics/Benzenoids	O
**107.0852**	o-Xylene	Aromatic hydrocarbons & solvents	O
**109.1007**	4-Vinylcyclohexene	Aromatic hydrocarbons & solvents	
**111.044**	5-Methylfurfural	Furan derivatives	O
**112.0507**	Cytosine	Nucleobases/Nucleosides & Cofactors	O
**112.0758**	1-Vinyl-2-pyrrolidone	Alkaloids & N-heterocycles	
**114.0552**	1-pyrroline-5-carboxylic acid	Amino acids & (cyclic) peptides	O
**114.0915**	Caprolactam	Alkaloids & N-heterocycles	O
**115.0502**	MFCD00021722	Amino acids & (cyclic) peptides	O
**115.0867**	1-Nitrosopiperidine	Alkaloids & N-heterocycles	O
**117.0546**	3-oxovaleric acid	Fatty acids & lipids	
**117.0912**	Butyl acetate	Fatty acids & lipids	O
**118.0653**	Benzyl cyanide	Phenolics/Benzenoids	O

**118.0654**	Indole	Indole-related	O
**119.085**	α-methylstyrene	Phenolics/Benzenoids	
**121.0645**	4-Vinylphenol	Phenolics/Benzenoids	O
**121.0649**	4-Methylbenzaldehyde	Phenolics/Benzenoids	O
**122.0967**	Phenethylamine	Alkaloids & N-heterocycles	O
**127.1117**	6-Methylhept-5-en-2-one	Sterols/Terpenoids & isoprenoids	O
**128.0708**	D-1-Piperideine-2-carboxylic acid	Amino acids & (cyclic) peptides	O
**129.0548**	2-Hydroxy-cis-hex-2,4-dienoate	Fatty acids & lipids	O
**129.0549**	Dihydrophloroglucinol	Phenolics/Benzenoids	O
**129.0913**	4-Methyl-3-methylenepentanoic acid	Fatty acids & lipids	
**129.1017**	D-Lysine lactam	Amino acids & (cyclic) peptides	O
**130.0647**	Isoquinoline	Alkaloids & N-heterocycles	O
**132.0805**	Skatole	Indole-related	O
**132.0808**	O-Methylbenzyl cyanide	Phenolics/Benzenoids	O
**133.1013**	Dicyclopentadiene	Fatty acids & lipids	
**134.0601**	4-indolol	Indole-related	
**134.0961**	(1R)-2-Phenylcyclopropanamine	Alkaloids & N-heterocycles	
**135.1163**	CY9070000	Phenolics/Benzenoids	
**135.1163**	Sec-Butylbenzene	Phenolics/Benzenoids	
**135.1167**	Durene	Phenolics/Benzenoids	
**136.0621**	Adenine	Nucleobases/Nucleosides & Cofactors	O
**136.0752**	(Z)-phenylacetaldoxime	Phenolics/Benzenoids	O

**136.1118**	Amphetamine	Nucleobases/Nucleosides & Cofactors	
**136.1123**	2,4,5-Trimethylaniline	Phenolics/Benzenoids	
**137.0463**	2H-pyrazolo[4,3-d]pyrimidin-7-ol	Alkaloids & N-heterocycles	
**137.0964**	4-Propylphenol	Phenolics/Benzenoids	
**137.1322**	(−)-β-Phellandrene	Sterols/Terpenoids & isoprenoids	O
**138.0551**	Anthranilic acid	Fatty acids & lipids	O
**138.0552**	Salicylamide	Fatty acids & lipids	
**138.0554**	Methyl nicotinate	Alkaloids & N-heterocycles	O
**138.0913**	4-(1-Aminoethyl)phenol	Phenolics/Benzenoids	
**139.0867**	4-Hydrazinobenzyl alcohol	Phenolics/Benzenoids	
**139.0871**	Propam	Fatty acids & lipids	
**139.1124**	3,6-Nonadienal	Nucleobases/Nucleosides & Cofactors	O
**140.1071**	(S)-Supinidine	Fatty acids & lipids	O
**142.0865**	L-α-Amino-β-(methylenecyclopropyl)propionic acid	Amino acids & (cyclic) peptides	
**143.1065**	2-propyl-4-pentenoic acid	Fatty acids & lipids	
**144.0479**	Sulfurol	Fatty acids & lipids	O
**146.0603**	VC4070000	Alkaloids & N-heterocycles	
**147.0658**	(R)-3-hydroxy-3-methyl-2-oxopentanoic acid	Fatty acids & lipids	O
**148.1118**	Actinidine	Alkaloids & N-heterocycles	O
**149.1073**	Nornicotine	Alkaloids & N-heterocycles	O
**149.1321**	Ectocarpene	Sterols/Terpenoids & isoprenoids	
**150.0392**	(3R)-3-HYDROXYASPARTATE	Amino acids & (cyclic) peptides	

**150.0552**	Gentianadine	Alkaloids & N-heterocycles	
**150.0778**	1-Methyladenine	Phenolics/Benzenoids	
**150.0781**	6-Methyladenine	Nucleobases/Nucleosides & Cofactors	
**150.1273**	2,6-Diethylaniline	Phenolics/Benzenoids	
**150.1284**	Phentermine	Alkaloids & N-heterocycles	
**152.0573**	Guanine	Nucleobases/Nucleosides & Cofactors	O
**152.1075**	N-Methyltyramine	Alkaloids & N-heterocycles	
**155.01**	Propanoyl phosphate	Fatty acids & lipids	
**155.0106**	Glycerol 1,2-cyclic phosphate	Fatty acids & lipids	
**158.0599**	1-Nitrosonaphthalene	Phenolics/Benzenoids	
**159.0438**	1,2-Naphthalenedione	Phenolics/Benzenoids	
**159.0769**	4-Methyleneglutamine	Amino acids & (cyclic) peptides	
**159.0771**	Oxiracetam	Alkaloids & N-heterocycles	
**160.0758**	Echinopsine	Fatty acids & lipids	
**160.0759**	3-methyl-2-quinolone	Alkaloids & N-heterocycles	O
**161.0598**	1-formylindan-2-one	Phenolics/Benzenoids	
**161.0601**	2-formylindan-1-one	Phenolics/Benzenoids	
**162.0549**	3-Carboxyindole	Indole-related	O
**162.0551**	2,4-Dihydroxyquinoline	Alkaloids & N-heterocycles	O
**163.0863**	2-Amino-5-phenyl-2-oxazoline	Alkaloids & N-heterocycles	
**163.123**	(S)-(-)-Anabasine	Alkaloids & N-heterocycles	O
**166.05**	2-Hydroxy-1,4-benzoxazin-3-one	Alkaloids & N-heterocycles	O

**166.0727**	Epiguanine	Nucleobases/Nucleosides & Cofactors	O
**166.0863**	L-(-)-Phenylalanine	Amino acids & (cyclic) peptides	O
**167.034**	4-hydroxyphenylglyoxylate	Phenolics/Benzenoids	
**167.0569**	Heteroxanthine	Nucleobases/Nucleosides & Cofactors	O
**169.0609**	2-Amino-4-nitroanisole	Phenolics/Benzenoids	
**169.0758**	β-Carboline; 9H-Pyrido[3,4-b]indole	Alkaloids & N-heterocycles	
**169.0971**	Pyridoxamine	Nucleobases/Nucleosides & Cofactors	O
**170.0969**	N-Phenylbenzenamine	Phenolics/Benzenoids	
**170.1541**	(6S,7R)-2-Azaspiro[5.5]undecan-7-ol	Fatty acids & lipids	
**170.9953**	(R)-2-O-sulfolactic acid	Fatty acids & lipids	
**172.1124**	(R)-N-Propargyl-1-aminoindan	Alkaloids & N-heterocycles	
**173.1025**	(2S,3R)-2-Amino-3-guanidinobutyric acid	Amino acids & (cyclic) peptides	
**173.1065**	Deoxypeganine	Fatty acids & lipids	O
**174.0553**	4-Quinolinecarboxylic acid	Alkaloids & N-heterocycles	
**174.0912**	Pyroquilon	Alkaloids & N-heterocycles	
**174.0918**	1,3-Dimethyl-8(2H)-isoquinolinone	Alkaloids & N-heterocycles	O
**174.1276**	1-Methyl-4-phenyl-1,2,3,6-tetrahydropyridine	Alkaloids & N-heterocycles	
**175.0067**	4-phenolsulfonic acid	Fatty acids & lipids	
**176.0707**	3-indoleglycolaldehyde	Indole-related	
**176.9706**	Medronic Acid	Fatty acids & lipids	
**177.055**	Hymecromone	Phenolics/Benzenoids	O
**177.0554**	Herniarin	Phenolics/Benzenoids	O

**177.0661**	Pemoline	Fatty acids & lipids	
**179.1423**	4-Hexylphenol	Phenolics/Benzenoids	
**180.0884**	7-Aminomethyl-7-deazaguanine	Nucleobases/Nucleosides & Cofactors	O
**180.102**	β-Phenyl-γ-aminobutyric acid	Amino acids & (cyclic) peptides	
**180.113**	Iproniazid	Alkaloids & N-heterocycles	
**181.0971**	(4-Ethoxyphenyl)urea	Alkaloids & N-heterocycles	
**181.1329**	Rilmenidine	Alkaloids & N-heterocycles	
**181.1585**	(3Z,6Z,9Z)-dodecatrienol	Sterols/Terpenoids & isoprenoids	
**182.1902**	Dicyclohexylamine	Alkaloids & N-heterocycles	
**183.0919**	Harmane	Alkaloids & N-heterocycles	
**183.1019**	[(R)-2,2,3-trimethyl-5-oxocyclopent-3-en-1-yl]acetic acid	Fatty acids & lipids	
**183.1377**	Decahydro-2-naphthalenecarboxylic acid	Fatty acids & lipids	
**184.007**	Saccharin	Fatty acids & lipids	
**184.0187**	Acephate	Fatty acids & lipids	
**184.1699**	(2,7-Dimethyloctahydro-1H-cyclopenta[c]pyridin-4-yl)methanol	Fatty acids & lipids	
**185.0921**	Veronal	Nucleobases/Nucleosides & Cofactors	
**185.1076**	Diphenyline	Unclassified/other	
**185.1283**	Apronalide	Unclassified/other	
**187.0866**	Credazine	Alkaloids & N-heterocycles	
**187.0866**	9-Oxodeoxypeganine	Alkaloids & N-heterocycles	O
**188.1095**	Molinate	Fatty acids & lipids	
**189.1022**	Phenazone	Alkaloids & N-heterocycles	

**190.0864**	Methyl 3-indolylacetate	Indole-related	
**192.1052**	1-methyl-3-phenyl-2,5-pyrrolidinedione	Amino acids & (cyclic) peptides	
**192.138**	Phendimetrazine	Alkaloids & N-heterocycles	
**193.0973**	2,6-Dihydroxy-N-methylmyosmine	Fatty acids & lipids	
**193.1579**	4-Heptylphenol	Phenolics/Benzenoids	
**195.0622**	Temozolomide	Nucleobases/Nucleosides & Cofactors	
**195.0765**	Aminohippuric acid	Fatty acids & lipids	
**195.1133**	pilocarpidine	Fatty acids & lipids	
**195.1743**	Dihydro-β-ionone	Sterols/Terpenoids & isoprenoids	
**196.1437**	Dolichotheline	Alkaloids & N-heterocycles	O
**197.1282**	Fasoracetam	Alkaloids & N-heterocycles	
**199.0723**	Mimosine	Alkaloids & N-heterocycles	
**199.1221**	Tacrine	Nucleobases/Nucleosides & Cofactors	
**199.1435**	(1S,6S)-6-Aminooctahydro-1-indolizinyl acetate	Indole-related	
**199.1477**	Guaiazulene	Sterols/Terpenoids & isoprenoids	
**199.1802**	Cycluron	Phenolics/Benzenoids	
**200.1182**	Pyrimethanil	Fatty acids & lipids	
**200.2012**	Lauramide	Fatty acids & lipids	
**201.0872**	Alanylclavam	Amino acids & (cyclic) peptides	
**201.1017**	Harmalol	Indole-related	
**202.0871**	Fenfuram	Alkaloids & N-heterocycles	
**202.1807**	11-Aminoundecanoic acid	Fatty acids & lipids	

**203.0638**	α-Naphthylthiourea	Alkaloids & N-heterocycles	
**204.1248**	3-(3-Methyl-2-buten-1-yl)-3H-purin-6-amine	Nucleobases/Nucleosides & Cofactors	
**204.1382**	2E-Crotamiton	Fatty acids & lipids	
**205.0863**	(Z)-3-butylidene-7-hydroxyphthalide	Phenolics/Benzenoids	O
**205.0973**	DL-Tryptophan	Indole-related	O
**205.1005**	Dihydroaceanthrylene	Phenolics/Benzenoids	
**205.1213**	Plastoquinone-1	Fatty acids & lipids	
**205.1944**	(E)-gamma-bisabolene	Sterols/Terpenoids & isoprenoids	
**205.1948**	Germacrene D	Sterols/Terpenoids & isoprenoids	
**205.195**	(S)-β-macrocarpene	Fatty acids & lipids	
**205.195**	α-Muurolene	Sterols/Terpenoids & isoprenoids	
**205.1956**	Thujopsene	Fatty acids & lipids	
**207.1127**	2-Ethyl-2-phenylmalonamide	Fatty acids & lipids	
**207.1348**	Actinamine	Alkaloids & N-heterocycles	
**208.1332**	Pentalamide	Fatty acids & lipids	
**209.1034**	(1R,2S)-1-(7,8-Dihydro-6-pteridinyl)-1,2-propanediol	Fatty acids & lipids	
**210.0763**	3-Carbamoyl-2-phenylpropionic acid	Fatty acids & lipids	
**210.113**	Lactylphenetidin	Fatty acids & lipids	
**211.0868**	AMAC	Phenolics/Benzenoids	
**211.1088**	1-(6-hydroxy-3-pyridyl)-4-(methylamino)butan-1-one	Alkaloids & N-heterocycles	
**211.1235**	Naphazoline	Alkaloids & N-heterocycles	
**212.1077**	Mebenil	Fatty acids & lipids	

**212.1393**	ISOXYL	Fatty acids & lipids	
**212.1641**	Elaeokanine C	Alkaloids & N-heterocycles	O
**213.1029**	5-Methyl-5,10-dihydro-1-phenazinol	Nucleobases/Nucleosides & Cofactors	
**213.1051**	Albutoin	Alkaloids & N-heterocycles	
**213.1139**	2-AMINO-3,4-DIMETHYLIMIDAZO(4,5-F)QUINOLINE	Alkaloids & N-heterocycles	O
**213.1232**	Butobarbital	Nucleobases/Nucleosides & Cofactors	
**213.1391**	Atipamezole	Fatty acids & lipids	
**213.1492**	Putaminoxin	Alkaloids & N-heterocycles	
**213.1596**	Brivaracetam	Alkaloids & N-heterocycles	
**215.106**	3,4′-Dihydroxybibenzyl	Phenolics/Benzenoids	O
**215.1175**	Fenyramidol	Alkaloids & N-heterocycles	
**215.1384**	Desthiobiotin	Nucleobases/Nucleosides & Cofactors	O
**216.1597**	Quwenzhi	Unclassified/other	
**217.0145**	5-(3-buten-1-ynyl)-2,2'-bithiophene	Phenolics/Benzenoids	
**217.0815**	8-Hydroxyalanylclavam	Amino acids & (cyclic) peptides	
**217.1332**	1-(4-hydroxyphenyl)-2-(piperidin-1-yl)acetonitrile	Alkaloids & N-heterocycles	O
**217.159**	Furanodiene	Sterols/Terpenoids & isoprenoids	O
**218.1037**	Pymetrozine	Alkaloids & N-heterocycles	
**218.118**	PYRACARBOLID	Alkaloids & N-heterocycles	
**219.1129**	N-Acetylserotonin	Indole-related	
**219.1134**	Rogletimide	Alkaloids & N-heterocycles	
**219.1339**	D-lysopine	Fatty acids & lipids	

**219.1343**	Meprobamate	Alkaloids & N-heterocycles	
**219.1739**	Germacrone	Sterols/Terpenoids & isoprenoids	O
**219.2104**	4,8,12-trimethyltrideca-1,3,7,11-tetraene	Sterols/Terpenoids & isoprenoids	O
**220.0838**	Uredepa	Fatty acids & lipids	
**220.1326**	Encyprate	Fatty acids & lipids	
**220.1696**	Fabianine	Alkaloids & N-heterocycles	O
**220.1699**	Sedamine	Alkaloids & N-heterocycles	
**221.0711**	Canthin-6-one	Alkaloids & N-heterocycles	O
**221.1534**	Glutinosone	Sterols/Terpenoids & isoprenoids	
**221.1899**	Alismol	Fatty acids & lipids	
**221.1901**	8-hydroxy-α-humulene	Sterols/Terpenoids & isoprenoids	
**222.1131**	Metaxalone	Alkaloids & N-heterocycles	
**222.1485**	Exalamide	Fatty acids & lipids	
**223.0645**	2-(3-methylthiopropyl)malic acid	Fatty acids & lipids	
**223.2047**	kunzeaol	Sterols/Terpenoids & isoprenoids	
**223.2053**	1-epi-Cubenol	Fatty acids & lipids	
**225.0919**	Isoflavone	Phenolics/Benzenoids	O
**225.1962**	Anapheline	Alkaloids & N-heterocycles	O
**225.2218**	DIHYDROFARNESOL	Sterols/Terpenoids & isoprenoids	
**226.0714**	2-amino-2-deoxyisochorismic acid	Fatty acids & lipids	
**226.0965**	Drometrizole	Phenolics/Benzenoids	

**226.1223**	2-[2-(4-Pyridinyl)-1-butenyl]phenol	Phenolics/Benzenoids	
**227.1181**	Metyrapone	Alkaloids & N-heterocycles	
**227.1286**	Butopyronoxyl	Fatty acids & lipids	
**227.1393**	Amobarbital	Nucleobases/Nucleosides & Cofactors	
**228.1958**	Cyprodenate	Fatty acids & lipids	
**229.0974**	Ozagrel	Fatty acids & lipids	
**229.2164**	Myristic acid	Fatty acids & lipids	O
**231.1138**	Methomidate	Fatty acids & lipids	
**232.0149**	N(ω)-Phosphohypotaurocyamine	Alkaloids & N-heterocycles	
**233.1279**	Aminoglutethimide	Alkaloids & N-heterocycles	
**233.1499**	Mebutamate	Fatty acids & lipids	
**233.2263**	Aplotaxene	Fatty acids & lipids	
**233.2264**	Gonane	Sterols/Terpenoids & isoprenoids	
**234.1852**	Faxeladol	Alkaloids & N-heterocycles	
**235.0755**	1,8-Pyrenediol	Phenolics/Benzenoids	
**235.1193**	Epirizole	Fatty acids & lipids	
**235.1324**	Strigolactone ABC-rings	Sterols/Terpenoids & isoprenoids	O
**235.1694**	Bakkenolide A	Sterols/Terpenoids & isoprenoids	O
**236.1652**	Phenol, 3-(2-(1,1-dimethylethyl)-3-methyl-5-oxazolidinyl)	Phenolics/Benzenoids	
**237.0663**	11-hydroxycanthin-6-one	Alkaloids & N-heterocycles	O
**237.1116**	3-Dimethylallyl-4-hydroxymandelic acid	Fatty acids & lipids	
**237.1132**	N,N′-(2,5-cyclohexadiene-1,4-diylidene)bis(1H-pyrrol-1-amine)	Alkaloids & N-heterocycles	

**237.2209**	(10E,12Z)-10,12-Hexadecadienal	Fatty acids & lipids	O
**239.034**	(3R)-3-Acetamido-3-methoxy-2-oxo-1-azetidinesulfonic acid	Fatty acids & lipids	
**239.1491**	Pirimicarb	Fatty acids & lipids	
**239.2363**	Bombykol	Fatty acids & lipids	O
**240.1031**	5-Hydroxy-2-acetamidofluorene	Phenolics/Benzenoids	
**240.1385**	N,N-dimethyl-2,2-diphenylacetamide	Phenolics/Benzenoids	
**241.1084**	2-(Hydroxyamino)-1-methyl-6-phenylimidazo[4,5-b]pyridine	Alkaloids & N-heterocycles	
**241.1339**	Ameltolide	Fatty acids & lipids	
**242.1745**	Valeroidine	Alkaloids & N-heterocycles	O
**243.0881**	Lumichrome	Nucleobases/Nucleosides & Cofactors	
**243.0977**	β-L-2'-deoxythymidine	Nucleobases/Nucleosides & Cofactors	
**243.1134**	N-(2,6-dimethylphenyl)-pyridine-3-carboxamide	Alkaloids & N-heterocycles	
**243.1376**	4,4a,5,6,7,8-Hexahydro-6-(p-hydroxyphenyl)-2(3H)- naphthalenone	Phenolics/Benzenoids	
**243.1488**	Huperzine	Fatty acids & lipids	
**244.2062**	Phencyclidine	Alkaloids & N-heterocycles	
**245.0956**	Hexahydro-2-oxo-1H-thieno[3,4-d]imidazole-4-valeric acid	Nucleobases/Nucleosides & Cofactors	
**245.1276**	Cyclo (L-Phe-L-Pro)	Amino acids & (cyclic) peptides	
**245.1286**	5-Oxo-N-(2-phenylcyclopropyl) prolinamide	Fatty acids & lipids	
**248.1387**	Parbendazole	Fatty acids & lipids	
**248.1503**	(1S)-1-Carboxy-2-(1H-indol-3-yl)-N,N,N- trimethylethanaminium	Indole-related	
**248.2012**	Lycopodine	Alkaloids & N-heterocycles	
**249.0497**	Furylfuramide	Fatty acids & lipids	

**249.1113**	Pyriculol	Phenolics/Benzenoids	
**249.1116**	3-dimethylallyl-4-hydroxyphenylpyruvic acid	Fatty acids & lipids	
**249.124**	6-Hydroxymelatonin	Indole-related	
**250.0869**	Dubamine	Alkaloids & N-heterocycles	
**251.1184**	Glycophymoline	Alkaloids & N-heterocycles	
**251.1506**	Verofylline	Nucleobases/Nucleosides & Cofactors	
**251.1643**	Piperdial	Fatty acids & lipids	
**251.1742**	3-Hydroxylidocaine	Alkaloids & N-heterocycles	
**251.2364**	12-(2-Cyclopenten-1-yl)-2-dodecanone	Fatty acids & lipids	
**252.1094**	2'-Deoxyadenosine	Nucleobases/Nucleosides & Cofactors	O
**253.1058**	Trinexapac -ethyl	Fatty acids & lipids	
**253.2531**	(9Z)-9-Cycloheptadecen-1-ol	Fatty acids & lipids	
**254.084**	Thiorphan	Fatty acids & lipids	
**254.1901**	Tolpropamine	Fatty acids & lipids	
**255.0654**	Primetin	Phenolics/Benzenoids	
**255.1604**	Ferimzone	Alkaloids & N-heterocycles	
**256.1337**	1-Methyl-6-(1,2,3,4-tetrahydro-6-hydroxy-2-naphthyl)-2(1H)-pyridone	**Alkaloids & N-heterocycles**	
**257.1291**	Ancymidol	Phenolics/Benzenoids	
**257.2466**	Palmitic Acid	Fatty acids & lipids	O
**258.1133**	TOLMETIN	Fatty acids & lipids	
**259.1447**	Cyclo (dehydrophenylalanyl-L-leucyl)	Amino acids & (cyclic) peptides	
**260.2001**	Eperisone	Fatty acids & lipids	

**260.2369**	(Z,Z,Z)-octadeca-4,7,10-trienenitrile	Fatty acids & lipids	
**261.1233**	Maculosin	Amino acids & (cyclic) peptides	
**261.1421**	Methaphenilene	Alkaloids & N-heterocycles	
**261.1481**	1-(4-Hydroxyphenyl)-1-decene-3,5-dione	Phenolics/Benzenoids	
**261.2581**	Androstane	Sterols/Terpenoids & isoprenoids	
**262.2159**	Cryptophorine	Alkaloids & N-heterocycles	
**263.1072**	1,6-Dimethoxypyrene	Phenolics/Benzenoids	
**263.2357**	4-Dodecylphenol	Phenolics/Benzenoids	
**265.1196**	Phenylacetylglutamine	Amino acids & (cyclic) peptides	
**266.1242**	LOBUCAVIR	Nucleobases/Nucleosides & Cofactors	
**266.1749**	Oxprenolol	Alkaloids & N-heterocycles	
**267.1849**	Desipramine	Alkaloids & N-heterocycles	
**268.1042**	Adenosine	Nucleobases/Nucleosides & Cofactors	O
**268.1549**	Codonopsine	Fatty acids & lipids	
**269.1284**	6-Methyl-9,10-didehydroergoline-8-carboxylic acid	Alkaloids & N-heterocycles	
**269.2476**	Cyclohexylundecanoic acid	Fatty acids & lipids	
**270.1125**	α-(4-methoxyphenyl)-6-methyl-2-pyridineacrylic acid	Alkaloids & N-heterocycles	
**270.1339**	Endralazine	Alkaloids & N-heterocycles	
**270.1476**	Strobamine	Alkaloids & N-heterocycles	
**270.2792**	Capsi -amide	Fatty acids & lipids	
**271.1693**	17β-Trenbolone	Sterols/Terpenoids & isoprenoids	
**271.2266**	16-Oxohexadecanoic acid	Fatty acids & lipids	

**273.2578**	(1E,3Z,6E,10E)-14-Isopropyl-3,7,11-trimethyl-1,3,6,10-cyclotetradecatetraene	Fatty acids & lipids	
**274.119**	Naphazoline nitrate	Alkaloids & N-heterocycles	
**274.2731**	Hexadecasphinganine	Sterols/Terpenoids & isoprenoids	
**275.2001**	Nandrolone	Sterols/Terpenoids & isoprenoids	O
**276.1457**	cipamfylline	Fatty acids & lipids	
**276.17**	(-)-Physostigmine	Indole-related	O
**277.1186**	Oxagrelate	Indole-related	O
**277.1701**	mebhydrolin	Alkaloids & N-heterocycles	
**277.1784**	buddledin A	Phenolics/Benzenoids	
**277.1798**	Sugeonyl acetate	Fatty acids & lipids	
**277.1805**	2-[(2E)-3,7-Dimethyl-2,6-octadien-1-yl]-6-methoxy-1,4-benzenediol	Phenolics/Benzenoids	
**277.2156**	Stearidonic acid	Fatty acids & lipids	
**277.2159**	Kinoprene	Sterols/Terpenoids & isoprenoids	O
**278.0245**	Fenitrothion	Phenolics/Benzenoids	
**278.1749**	promacyl	Phenolics/Benzenoids	
**279.1498**	azanator	Alkaloids & N-heterocycles	
**279.186**	Triprolidine	Alkaloids & N-heterocycles	
**279.2313**	γ-Linolenic acid	Fatty acids & lipids	O
**279.2318**	α-Linolenic acid	Fatty acids & lipids	O
**279.2329**	5β-estran-3α,17β-diol	Sterols/Terpenoids & isoprenoids	
**280.1446**	Ramosetron	Alkaloids & N-heterocycles	
**280.1899**	Serratine	Alkaloids & N-heterocycles	

**281.1288**	cyclopeptine	Fatty acids & lipids	
**281.2476**	Malvalic acid	Fatty acids & lipids	
**281.2477**	Chaulmoogric Acid	Fatty acids & lipids	
**282.1198**	1-Methyladenosine	Nucleobases/Nucleosides & Cofactors	O
**282.2203**	Alverine	Alkaloids & N-heterocycles	
**282.2786**	Laurocapram	Fatty acids & lipids	
**282.2793**	(9Z)-9-Octadecenamide	Fatty acids & lipids	
**283.1696**	miltirone	Sterols/Terpenoids & isoprenoids	
**283.2635**	Petroselinic acid	Fatty acids & lipids	
**284.0992**	Guanosine	Nucleobases/Nucleosides & Cofactors	O
**284.1393**	Cyclo (L-Trp-L-Pro)	Amino acids & (cyclic) peptides	O
**286.11**	Letrozole	Alkaloids & N-heterocycles	
**286.1448**	Apohyoscine	Alkaloids & N-heterocycles	
**287.139**	Schradan	Alkaloids & N-heterocycles	
**287.1616**	Abacavir	Nucleobases/Nucleosides & Cofactors	
**289.1071**	Asebogenin	Phenolics/Benzenoids	O
**289.1189**	APAZIQUONE	Fatty acids & lipids	
**290.2474**	17α-Aza-D-homoandrost-5-en-3β-ol	Amino acids & (cyclic) peptides	
**291.1855**	Azatadine	Alkaloids & N-heterocycles	
**291.1949**	ML-236C	Sterols/Terpenoids & isoprenoids	O
**291.2272**	Succinylcholine	Amino acids & (cyclic) peptides	
**292.1807**	Perlapine	Alkaloids & N-heterocycles	

**293.1293**	N-(2-methoxyphenyl)-N′-2-naphthalenyl-urea	Alkaloids & N-heterocycles	
**293.165**	Cinchonidinone	Alkaloids & N-heterocycles	O
**293.1795**	Thioperamide	Alkaloids & N-heterocycles	
**294.1231**	1-(4-Aminophenyl)-4-methyl-7,8-methylenedioxy-5H-2,3-benzodiazepine	Alkaloids & N-heterocycles	
**294.1303**	Pimethixene	Fatty acids & lipids	
**294.2065**	Dihydrobunolol	Alkaloids & N-heterocycles	
**295.1445**	2-(3-Ethyl-5-(4-methoxyphenyl)-1H-pyrazol-4-yl)phenol	Phenolics/Benzenoids	
**295.1547**	Alosetron	Alkaloids & N-heterocycles	
**295.1897**	Embelin	Phenolics/Benzenoids	
**296.1356**	N6-Dimethyladenosine	Nucleobases/Nucleosides & Cofactors	O
**296.1416**	Sumatriptan	Alkaloids & N-heterocycles	
**296.1756**	Dibenzepin	Fatty acids & lipids	
**296.2367**	Diisopromine	Alkaloids & N-heterocycles	
**297.1272**	Glycyclamide	Fatty acids & lipids	
**297.1346**	Rosin	Phenolics/Benzenoids	O
**297.1581**	Guanethidine monosulfate	Fatty acids & lipids	
**298.115**	PSICOFURANINE	Fatty acids & lipids	
**298.2727**	(-)-cassine	Alkaloids & N-heterocycles	O
**298.2738**	3-dehydrosphingosine	Fatty acids & lipids	
**299.0816**	5'-S-Methyl-5'-thioinosine	Nucleobases/Nucleosides & Cofactors	
**299.084**	mefenacet [JMAF]	Alkaloids & N-heterocycles	
**299.142**	2-(3-Ethyl-4-oxo-5-piperidino-2-thiazolidinylidene)acetic acid ethyl ester	Fatty acids & lipids	

**299.2576**	18-hydroxyoleic acid	Fatty acids & lipids	
**299.2577**	RG1300000	Unclassified/other	
**299.332**	2-Octyl-1-dodecanol	Fatty acids & lipids	O
**300.2892**	3-ketosphinganine	Fatty acids & lipids	
**301.0754**	sulfaquinoxaline	Unclassified/other	
**302.1962**	Tegaserod	Alkaloids & N-heterocycles	
**302.3055**	L-threo-dihydrosphingosine	Fatty acids & lipids	O
**304.2629**	fenpropimorph	Fatty acids & lipids	
**305.161**	Balfourodinine	Alkaloids & N-heterocycles	O
**305.2007**	kevopril	Alkaloids & N-heterocycles	
**305.2013**	CP-339818	Alkaloids & N-heterocycles	
**306.1649**	buprofezin	Alkaloids & N-heterocycles	
**307.1296**	Z-Gly-Pro	Amino acids & (cyclic) peptides	
**307.1772**	feruloylagmatine	Fatty acids & lipids	
**307.1899**	6-Desmethylmonacolin J	Fatty acids & lipids	
**307.2625**	cembra-2,7,11-triene-4,6-diol	Sterols/Terpenoids & isoprenoids	
**308.149**	8-Hydroxy-8-azabicyclo[3.2.1]oct-3-yl 3,4-dimethoxybenzoate	Phenolics/Benzenoids	
**308.1647**	Moxaverine	Alkaloids & N-heterocycles	
**308.1751**	Talastine	Alkaloids & N-heterocycles	
**308.2577**	Pumiliotoxin A	Sterols/Terpenoids & isoprenoids	O
**308.2582**	Fingolimod	Alkaloids & N-heterocycles	
**309.1318**	14-Dihydroxycornestin	Phenolics/Benzenoids	O

**309.1697**	Inulicin	Sterols/Terpenoids & isoprenoids	O
**309.2073**	Soraphen O	Sterols/Terpenoids & isoprenoids	O
**310.114**	N-Acetylneuraminic acid	Fatty acids & lipids	O
**310.1148**	9-O-acetylneuraminic acid	Fatty acids & lipids	O
**310.2744**	Dicyclomine	Alkaloids & N-heterocycles	
**311.055**	6-demethylsterigmatocystin	Phenolics/Benzenoids	O
**311.0818**	Sulfadoxine	Nucleobases/Nucleosides & Cofactors	
**311.0861**	Acromelic acid A	Fatty acids & lipids	
**311.0917**	6-Deoxyjacareubin	Phenolics/Benzenoids	O
**311.14**	Aplindore	Fatty acids & lipids	
**311.1459**	Mannopine	Fatty acids & lipids	
**311.1743**	Raupine	Fatty acids & lipids	
**311.1748**	(8α,9R)-Cinchonan-6',9-diol	Alkaloids & N-heterocycles	O
**311.1853**	Botrydial	Sterols/Terpenoids & isoprenoids	O
**312.1347**	Imazaquin	Alkaloids & N-heterocycles	
**313.0705**	(5Z)-3-(2,4-Dihydroxyphenyl)-4-hydroxy-5-(4-hydroxybenzylidene)-2(5H)-furanone	Phenolics/Benzenoids	
**313.1481**	Olanzapine	Fatty acids & lipids	
**313.2358**	5,8-dihydroxy-octadecadienoic acid	Fatty acids & lipids	O
**314.072**	4-nitro-N-trichloromethylthio-phthalimide	Phenolics/Benzenoids	
**315.0685**	Rofecoxib	Alkaloids & N-heterocycles	
**316.1304**	anthramycin	Phenolics/Benzenoids	
**316.205**	Fendiline	Alkaloids & N-heterocycles	
**316.2115**	butoctamide	Fatty acids & lipids	

**316.2477**	C10-Carnitine	Fatty acids & lipids	
**317.1761**	1-Dehydro-15α-hydroxytestololactone	Phenolics/Benzenoids	
**317.2828**	17-Propyl-5α-androst-2-en-17β-ol	Sterols/Terpenoids & isoprenoids	
**318.2791**	Funtumine	Alkaloids & N-heterocycles	O
**318.2999**	Phytosphingosine	Fatty acids & lipids	
**319.2866**	Tridihexethyl	Alkaloids & N-heterocycles	
**320.1745**	1-carbazol-9-yl-3-(3,5-dimethyl-pyrazol-1-yl)-propan-2-ol	Indole-related	
**321.1601**	Feprazone	Indole-related	
**321.1703**	α-Zearalenol	Phenolics/Benzenoids	O
**321.206**	Testolic acid	Fatty acids & lipids	
**323.1749**	6'-Methoxycinchonan-9-one	Alkaloids & N-heterocycles	O
**323.1753**	Gardneral	Phenolics/Benzenoids	O
**323.2585**	2α-(Hydroxymethyl)-5α-androstane-3β,17β-diol	Sterols/Terpenoids & isoprenoids	
**324.1445**	Acremoauxin A	Sterols/Terpenoids & isoprenoids	O
**325.1753**	Tricetamide	Fatty acids & lipids	
**326.2111**	Ifenprodil	Alkaloids & N-heterocycles	
**327.0779**	Triphenyl phosphate	Phenolics/Benzenoids	
**327.0875**	Dihydrosterigmatocystin	Phenolics/Benzenoids	O
**327.1341**	cyclo (L-tyr-L-tyr)	Amino acids & (cyclic) peptides	O
**328.1914**	Difemerine	Alkaloids & N-heterocycles	
**328.3208**	N,N-Dimethylsphingosine	Fatty acids & lipids	
**329.1099**	7-Hydroxy-6-methyl-8-(1-D-ribityl)lumazine	Nucleobases/Nucleosides & Cofactors	

**329.1897**	4-(3,5-Diphenylcyclohexyl)phenol	Phenolics/Benzenoids	
**330.1896**	Gabapentin enacarbil	Alkaloids & N-heterocycles	
**331.145**	Gambirtannine	Alkaloids & N-heterocycles	O
**333.1914**	(2S,3S)-2-hydroxytridecane-1,2,3-tricarboxylic acid	Fatty acids & lipids	
**333.2298**	3-[(3-Hydroxy-2-phenylpropanoyl)oxy]-8-isopropyl-8-methyl-8-azoniabicyclo[3.2.1]octane	Fatty acids & lipids	
**334.1503**	Salicylate Meglumine	Phenolics/Benzenoids	
**335.1151**	1-Hydroxy-2-methoxy-12-methyl[1,3]benzodioxolo[5,6-c]phenanthridin-12-ium	Phenolics/Benzenoids	
**335.1261**	Neostigmine Methylsulfate	Fatty acids & lipids	
**335.1766**	Vinorine	Alkaloids & N-heterocycles	O
**335.2219**	Resolvin E2	Fatty acids & lipids	O
**336.2528**	Oxeladin	Alkaloids & N-heterocycles	
**337.1533**	Cyclopiazonic acid	Fatty acids & lipids	
**337.1912**	Methyl 3,4-didehydroibogamine-18-carboxylate	Alkaloids & N-heterocycles	
**337.2365**	Leukotriene B4	Alkaloids & N-heterocycles	O
**338.1909**	Istamycin AO	Alkaloids & N-heterocycles	
**339.1789**	(6E)-8,9-Dihydroxy-2-oxo-10-propyl-3,4,5,8,9,10-hexahydro-2H-oxecin-3-yl (2E,4E)-2,4-hexadienoate	Fatty acids & lipids	
**339.2151**	Idebenone	Nucleobases/Nucleosides & Cofactors	O
**339.2687**	3-(2,4-Cyclopentadien-1-ylidene)-5α-androstan-17β-ol	Fatty acids & lipids	
**340.126**	Loxoribine	Nucleobases/Nucleosides & Cofactors	
**340.2848**	Rociverine	Alkaloids & N-heterocycles	
**341.3039**	Glycidyl Stearate	Fatty acids & lipids	
**342.2063**	Propafenone	Alkaloids & N-heterocycles	

**343.1689**	Penicillin A	Fatty acids & lipids	O
**344.3155**	Ethanolamine oleate	Alkaloids & N-heterocycles	
**345.1845**	α,α'-Diethyl-4,4'-bis(2-propynyloxy)stilbene	Phenolics/Benzenoids	
**346.3103**	Irehine	Alkaloids & N-heterocycles	O
**347.0849**	3'-Amino-3'-deoxyadenosine 5'-(dihydrogen phosphate)	Nucleobases/Nucleosides & Cofactors	O
**352.1615**	2-methyl-4-(1H-purin-6-ylamino)but-2-en-1-yl β-D- xylopyranoside	Nucleobases/Nucleosides & Cofactors	
**353.0504**	4-[2-(5-Carboxy-2-hydroxy-3-methoxyphenyl)-2-oxoethylidene]-2-hydroxy-2-pentenedioate	Fatty acids & lipids	
**353.176**	4-Ethyl-3-(4-methoxyphenyl)-2,2-dimethyl-2H-chromen-7-yl acetate	Fatty acids & lipids	
**353.248**	Quinbolone	Sterols/Terpenoids & isoprenoids	
**357.1617**	Hycanthone	Alkaloids & N-heterocycles	
**357.278**	Ethyl docosahexaenoate	Fatty acids & lipids	O
**358.1846**	Uplandicine	Fatty acids & lipids	
**358.1889**	Cafedrine	Alkaloids & N-heterocycles	
**359.2941**	Bufanolide	Sterols/Terpenoids & isoprenoids	O
**361.1573**	N-(4-(1-methyl-2-((isopropyl)sulfonamido)ethyl)phenyl)benzamide	Alkaloids & N-heterocycles	
**361.2364**	Iloprost	Fatty acids & lipids	O
**362.1696**	Acrinol	Phenolics/Benzenoids	
**362.1953**	Buquinolate	Fatty acids & lipids	
**362.3047**	CETABEN	Fatty acids & lipids	
**365.1947**	Resiniferonol	Fatty acids & lipids	
**367.1389**	1α,5α-Epidithio-17a-oxa-D-homoandrostan-3,17-dione	Amino acids & (cyclic) peptides	
**367.1703**	N-[(3-Hydroxy-4,7,7-trimethylbicyclo[2.2.1]hept-2-yl)carbamoyl]-4-methylbenzenesulfonamide	Fatty acids & lipids	

**367.2197**	(1R,2S,3S,4S,5S,6R)-2-Amino-3,4,6-trihydroxy-5-(methylamino)cyclohexyl (5R)-2-amino-5 -[(1S)-1-aminoethyl]-2-deoxy-α-D-xylopyranoside	Alkaloids & N-heterocycles	
**367.238**	(8R,9S)-fumigaclavine C	Indole-related	O
**367.3571**	Nervonic acid	Fatty acids & lipids	O
**370.1814**	Amisulpride	Alkaloids & N-heterocycles	
**371.2223**	Spiromesifen	Fatty acids & lipids	
**376.1395**	Tiagabine	Alkaloids & N-heterocycles	
**376.3203**	Docosatetraenoylethanolamide	Fatty acids & lipids	O
**377.2148**	17β-Hydroxy-7α-mercaptoandrost-4-en-3-one 7-propionate	Fatty acids & lipids	
**377.2693**	3-Acetyl-5α-androstane-3β,17β-diol 3-acetate	Fatty acids & lipids	
**380.1879**	sudan 7b	Phenolics/Benzenoids	
**381.1584**	LACTITOL DIHYDRATE	Phenolics/Benzenoids	
**381.2049**	Chlorophorin	Phenolics/Benzenoids	O
**383.2044**	Sumarotene	Sterols/Terpenoids & isoprenoids	O
**383.2929**	Quingestrone	Sterols/Terpenoids & isoprenoids	
**384.2537**	Naftidrofuryl	Alkaloids & N-heterocycles	
**385.1282**	S-Adenosyl-L-homocysteine	Nucleobases/Nucleosides & Cofactors	O
**385.1541**	(6E)-3,7,11-Trimethyl-6,10-dodecadien-1-yl trihydrogen diphosphate	Fatty acids & lipids	
**385.2535**	Ibutilide	Alkaloids & N-heterocycles	
**385.3463**	Desmesterol	Fatty acids & lipids	O
**388.2838**	Myxalamid C	Sterols/Terpenoids & isoprenoids	O
**389.2592**	Galeterone	Sterols/Terpenoids & isoprenoids	
**391.1472**	Regadenoson	Nucleobases/Nucleosides & Cofactors	

**391.1897**	6,8-Di-(3,3-dimethylallyl)chrysin	Phenolics/Benzenoids	O
**391.2843**	α-Apocholic acid	Fatty acids & lipids	O
**394.2358**	Oxidized Cypridina luciferin	Phenolics/Benzenoids	O
**397.2182**	Etarotene	Sterols/Terpenoids & isoprenoids	
**397.2747**	Estradiol cypionate	Fatty acids & lipids	
**397.3099**	δ-Tocotrienol	Sterols/Terpenoids & isoprenoids	O
**398.3415**	Solanidine	Sterols/Terpenoids & isoprenoids	O
**399.179**	N-benzoyl-D-arginine-4-nitroanilide	Amino acids & (cyclic) peptides	
**399.1807**	L-365,260	Alkaloids & N-heterocycles	
**399.3618**	(3S,5E,7E,10S,14xi,17xi,22E)-9,10-Secoergosta-5,7,22-trien-3-ol	Alkaloids & N-heterocycles	
**402.1343**	Oxyphenisatin Acetate	Fatty acids & lipids	
**402.2837**	Myriocin	Sterols/Terpenoids & isoprenoids	O
**403.2703**	Erythronolide B	Sterols/Terpenoids & isoprenoids	O
**404.3**	Spergualin	Fatty acids & lipids	
**407.1953**	Carvedilol	Alkaloids & N-heterocycles	
**409.383**	15-cis-4,4'-diapophytoene	Sterols/Terpenoids & isoprenoids	
**410.3047**	Veratramine	Alkaloids & N-heterocycles	
**411.2081**	Quide	Unclassified/other	
**411.237**	Nigaki Hemiacetal A	Sterols/Terpenoids & isoprenoids	O
**411.2732**	(3β)-Gonan-3-yl β-D-glucopyranoside	Sterols/Terpenoids & isoprenoids	
**411.29**	Norethisterone enanthate	Fatty acids & lipids	
**411.3606**	4α-methyl-5α-ergosta-8,14,24(28)-trien-3β-ol	Sterols/Terpenoids & isoprenoids	

**412.3198**	Cyclopamine	Fatty acids & lipids	O
**413.3772**	4α-Methylfecosterol	Sterols/Terpenoids & isoprenoids	
**413.3776**	Vitamin D5	Sterols/Terpenoids & isoprenoids	O
**413.3783**	Stigmasterol	Sterols/Terpenoids & isoprenoids	O
**413.3784**	(3β,4α,9xi,14xi,17xi)-4-Methylergosta-7,24(28)-dien-3-ol	Sterols/Terpenoids & isoprenoids	
**414.3365**	Solasodine	Sterols/Terpenoids & isoprenoids	O
**415.2008**	Naltrindole	Indole-related	
**415.3222**	(5β)-14-Hydroxycholest-7-ene-3,6-dione	Sterols/Terpenoids & isoprenoids	
**415.3573**	5α-ergosta-7,22-diene-3β,5-diol	Sterols/Terpenoids & isoprenoids	
**416.2804**	Bimatoprost	Fatty acids & lipids	
**416.3526**	Tomatidine	Sterols/Terpenoids & isoprenoids	
**417.2471**	2-[(2R,4aS,6R,7R,8aR)-6,7-Dihydroxy-4a-methyl-8-methylenedecahydro-2-naphthalenyl]-2-propanyl β-D-glucopyranoside	Fatty acids & lipids	
**417.2822**	Istamycin B3	Alkaloids & N-heterocycles	
**419.3158**	Maxacalcitol	Sterols/Terpenoids & isoprenoids	
**420.3099**	Myxalamid S	Sterols/Terpenoids & isoprenoids	O
**425.2154**	(3α,4β,8α,12R)-15-Acetoxy-3,4-dihydroxy-12,13-epoxytrichothec-9-en-8-yl 3-methylbutanoate	Fatty acids & lipids	
**425.3411**	α-Tocotrienol	Sterols/Terpenoids & isoprenoids	O
**426.262**	Piriprost	Fatty acids & lipids	
**427.2841**	Azafrin	Sterols/Terpenoids & isoprenoids	O
**427.302**	Leupeptin Ac-LL	Fatty acids & lipids	
**429.2115**	Bendazac L-lysine	Amino acids & (cyclic) peptides	
**429.3718**	(3β,24R,24'R)- fucosterol epoxide	Sterols/Terpenoids & isoprenoids	

**430.2436**	Melagatran	Alkaloids & N-heterocycles	
**430.3309**	Peiminine	Fatty acids & lipids	
**432.1457**	Tiodazosin	Alkaloids & N-heterocycles	
**433.2013**	Aspulvinone H	Phenolics/Benzenoids	O
**433.3785**	Solacapine	Fatty acids & lipids	
**435.259**	Elarofiban	Alkaloids & N-heterocycles	
**435.2873**	Elarofiban	Fatty acids & lipids	
**437.303**	Bolmantalate	Fatty acids & lipids	
**441.3378**	Pfaffic acid	Fatty acids & lipids	O
**444.223**	Dromoran	Alkaloids & N-heterocycles	O
**445.2939**	Fusaproliferin	Sterols/Terpenoids & isoprenoids	O
**446.2381**	Lomibuvir	Alkaloids & N-heterocycles	
**447.3465**	Stylisterol B	Sterols/Terpenoids & isoprenoids	O
**449.2702**	Isotretinoin anisatil	Sterols/Terpenoids & isoprenoids	
**451.1586**	Auriculoside	Sterols/Terpenoids & isoprenoids	O
**457.2311**	Methyl (2β,4β,5α,12β,19α)-4-acetoxy-3-hydroxy-16-methoxy-1-methyl-6,7-didehydroaspidospermidine-3-carboxylate	Fatty acids & lipids	
**467.3526**	PHYLLOQUINONE OXIDE	Sterols/Terpenoids & isoprenoids	
**468.3092**	(5α,14β,18R)-17-(Cyclopropylmethyl)-18 -[(2R)-2-hydroxy-3,3-dimethyl-2-butanyl]-6-methoxy-18,19-dihydro-4,5-epoxy-6,14-ethenomorphinan-3-ol	Alkaloids & N-heterocycles	O
**469.2689**	Ivabradine	Alkaloids & N-heterocycles	
**469.3316**	3-Oxoglycyrrhetic acid	Fatty acids & lipids	
**470.1488**	Bendacalol Mesylate	Fatty acids & lipids	
**473.1868**	5-Formiminotetrahydrofolic acid	Fatty acids & lipids	

**473.2568**	LY-170680	Alkaloids & N-heterocycles	
**476.3065**	Netilmicin	Alkaloids & N-heterocycles	
**481.3534**	Brassinolide	Sterols/Terpenoids & isoprenoids	
**484.3917**	Oxolucidine B	Phenolics/Benzenoids	O
**495.1274**	SALVIANOLIC ACID	Fatty acids & lipids	
**499.2168**	Iridodial glucoside tetraacetate	Fatty acids & lipids	
**500.3043**	Taurochenodeoxycholic acid	Fatty acids & lipids	
**503.2561**	Yazumycin A	Alkaloids & N-heterocycles	O
**504.28**	Mycalamide A	Fatty acids & lipids	
**506.2001**	Alangiside	Sterols/Terpenoids & isoprenoids	O
**529.3862**	Pachymic acid	Fatty acids & lipids	O
**531.4026**	α-Tocopheryl succinate	Sterols/Terpenoids & isoprenoids	O
**533.4359**	2-Hexaprenyl-6-methoxyphenol	Phenolics/Benzenoids	
**536.1043**	Lipoyl-AMP	Nucleobases/Nucleosides & Cofactors	O
**549.361**	Manzamine A	Alkaloids & N-heterocycles	O
**551.5021**	1-O-(1Z-Tetradecenyl)-2-(9Z-octadecenoyl)- sn -glycerol	Fatty acids & lipids	
**551.5059**	Octenidine	Alkaloids & N-heterocycles	
**555.2515**	Pantethine	Fatty acids & lipids	O
**563.3205**	Carpipramine maleate	Fatty acids & lipids	
**563.3342**	Tandutinib	Alkaloids & N-heterocycles	
**569.3139**	Crenatine A	Amino acids & (cyclic) peptides	O
**577.1335**	Procyanidin A2	Phenolics/Benzenoids	O

**579.417**	Okenone	Sterols/Terpenoids & isoprenoids	O
**585.2892**	Ouabain	Sterols/Terpenoids & isoprenoids	O
**589.1888**	Etoposide	Phenolics/Benzenoids	O
**594.1573**	Cefteram pivoxil	Fatty acids & lipids	
**619.4516**	Valomaciclovir stearate	Fatty acids & lipids	O
**631.114**	GDP-N-acetyl-α-D-perosamine	Fatty acids & lipids	O
**635.4136**	Melilotoside A2	Sterols/Terpenoids & isoprenoids	O
**656.1934**	Malvin	Phenolics/Benzenoids	O
**660.309**	Hydergine	Alkaloids & N-heterocycles	O
**671.3887**	Saquinavir	Alkaloids & N-heterocycles	
**675.4938**	1-Palmitoyl-2-oleoylphosphatidic acid	Fatty acids & lipids	O
**704.4377**	Spinosyn P	Fatty acids & lipids	O
**720.4319**	Carfilzomib	Alkaloids & N-heterocycles	
**749.5343**	1-PALMITOYL-2-OLEOYL-SN-GLYCERO-3-(PHOSPHO-RAC-(1-GLYCEROL))	Fatty acids & lipids	O
**751.6014**	Nonaprenyl 4-hydroxybenzoate	Phenolics/Benzenoids	O
**785.4653**	(3β,6α,9β,16β,20R,24S)-6,16,25-Trihydroxy-20,24-epoxy-9,19-cyclolanostan-3-yl 2-O-[(2S,3R,4R)-3,4-dihydroxy-4-(hydroxymethyl)tetrahydro-2-furanyl]-β-D-glucopyranoside	Sterols/Terpenoids & isoprenoids	O
**852.5102**	α-Chaconine	Sterols/Terpenoids & isoprenoids	O
**869.4898**	Desglucomusennin	Sterols/Terpenoids & isoprenoids	O
**955.7052**	(3R,3'R)-3-Hydroxy-β,β-caroten-3'-yl 6-O-(13-methyltetradecanoyl)-D-glucopyranoside	Fatty acids & lipids	O
